# Using Satellite Tracking to Optimize Protection of Long-Lived Marine Species: Olive Ridley Sea Turtle Conservation in Central Africa

**DOI:** 10.1371/journal.pone.0019905

**Published:** 2011-05-11

**Authors:** Sara M. Maxwell, Greg A. Breed, Barry A. Nickel, Junior Makanga-Bahouna, Edgard Pemo-Makaya, Richard J. Parnell, Angela Formia, Solange Ngouessono, Brendan J. Godley, Daniel P. Costa, Matthew J. Witt, Michael S. Coyne

**Affiliations:** 1 Ocean Sciences Department, University of California Santa Cruz, Santa Cruz, California, United States of America; 2 Department of Ecology and Evolutionary Biology, University of California Santa Cruz, Santa Cruz, California, United States of America; 3 Center for Integrated Spatial Research, University of California Santa Cruz, Santa Cruz, California, United States of America; 4 Wildlife Conservation Society, Parc National de Mayumba, Mayumba, Gabon; 5 Wildlife Conservation Society, Global Conservation Program, New York, New York, United States of America; 6 Agence Nationale des Parcs Nationaux, Libreville, Gabon; 7 Marine Turtle Research Group, Centre for Ecology and Conservation, University of Exeter, Penyrn, Cornwall, United Kingdom; 8 SEATURTLE.org, Durham, North Carolina, United States of America; Institut Pluridisciplinaire Hubert Curien, France

## Abstract

Tractable conservation measures for long-lived species require the intersection between protection of biologically relevant life history stages and a socioeconomically feasible setting. To protect breeding adults, we require knowledge of animal movements, how movement relates to political boundaries, and our confidence in spatial analyses of movement. We used satellite tracking and a switching state-space model to determine the internesting movements of olive ridley sea turtles (*Lepidochelys olivacea*) (n = 18) in Central Africa during two breeding seasons (2007-08, 2008-09). These movements were analyzed in relation to current park boundaries and a proposed transboundary park between Gabon and the Republic of Congo, both created to reduce unintentional bycatch of sea turtles in marine fisheries. We additionally determined confidence intervals surrounding home range calculations. Turtles remained largely within a 30 km radius from the original nesting site before departing for distant foraging grounds. Only 44.6 percent of high-density areas were found within the current park but the proposed transboundary park would incorporate 97.6 percent of high-density areas. Though tagged individuals originated in Gabon, turtles were found in Congolese waters during greater than half of the internesting period (53.7 percent), highlighting the need for international cooperation and offering scientific support for a proposed transboundary park. This is the first comprehensive study on the internesting movements of solitary nesting olive ridley sea turtles, and it suggests the opportunity for tractable conservation measures for female nesting olive ridleys at this and other solitary nesting sites around the world. We draw from our results a framework for cost-effective protection of long-lived species using satellite telemetry as a primary tool.

## Introduction

Protection of natural resources is a global priority, yet implementation of conservation measures in complex socio-political contexts is often challenging [1]–[3]. Tangible conservation measures for long-lived marine species requires that protection of biologically relevant life history stages be logistically, politically and economically feasible [4]–[6]. While studies have shown the vulnerability of early life stages of some marine species (e.g. sea turtles [7], [8], seabirds [9], elasmobranchs [10], seals [11]), protection of breeding adults of long-lived species sustains populations in two ways. First, breeding individuals contribute disproportionately to sustaining the population compared to non-breeding individuals [6], [12]. Second, for many species, reproductive activities take place in distinct geographic regions and span several months. Such discrete regions are often highly vulnerable, but allow practical protection that is more feasible than in cases where individuals are dispersed throughout the range [13], [14].

Sea turtles are both excellent candidates and models for protection of vulnerable, discrete breeding areas. Sea turtle nesting seasons usually span several months during which females return repeatedly to the same beach to lay a variable number of clutches [15]. Despite many species being highly migratory in other parts of their range, both male and female turtles return from distant foraging grounds and remain in the vicinity of the nesting beach for both mating and nesting and thus, reproductive individuals are aggregated in space and time [16]–[20]. As males and females have been shown to encompass similar areas due to related mating and nesting activities, protecting the range of breeding females is also likely to encompass male distributions [17]. Adequate protection of breeding females, however, requires knowledge of three key elements: (1) the movements of animals between nesting events; (2) how these movements relate to management and political boundaries; and (3) our level of confidence in the precision of inferred movements given the methods used relative to the spatial scale of analyses.

Internesting movements vary considerably between species and understanding these movements is critical for the first element of effective protection. Some loggerhead sea turtles (*Caretta caretta*) remain within a few kilometers of the original nest, while leatherbacks (*Dermochelys coriacea*) and green turtles (*Chelonia mydas*) can cover hundreds of kilometers between nests [21]–[25]. Thus, knowledge of the spatial and temporal scale of internesting movements dictates the scale at which protective measures are necessary, helping managers put management actions in better context of human and ecological needs. Satellite telemetry has proven an effective tool for gaining knowledge of at-sea behavior because it enables us to determine movements away from land and is especially useful on remote nesting beaches where turtles are not reencountered frequently [26].

The second element to successful protection is understanding how these movements relate to spatially-based management strategies such as marine protected areas (MPAs). MPAs are often used to protect sensitive species by reducing activities such as fishing within their boundaries, but are only effective if park boundaries are drawn to adequately incorporate all important areas used by the protected species, and if MPA boundaries can be adequately enforced [27]–[31]. MPAs designated without full knowledge of protected species distributions can unintentionally displace and concentrate fishing effort in unprotected areas of high use by the species they are intended to conserve [32].

Satellite telemetry has been proven to be an effective means of observing how animal biology and movement relate to political boundaries [25], [33]–[36] but this leads to the third critical element in adequate protection: our level of confidence in tracks given known limitations of our methodologies. The inherent error associated with satellite telemetry can reduce our confidence in location and density estimates. When areas under observation are on a small spatial scale relative to satellite location error, analyses and inferences can be negatively affected [37]–[39] and could result in protective boundaries such as MPAs being placed ineffectually. New advances in the processing of satellite telemetry datasets, however, allow us to robustly consider observation error. State-space models separate observation error from behavioral processes in analysis of animal movements. This allows researchers to estimate confidence intervals at each location and better estimate biologically relevant parameters [38]. These confidence intervals can then be used to inform subsequent spatial analyses, allowing us to consider this error when recommending conservation measures such as the position of park boundaries.

**Figure 1:**
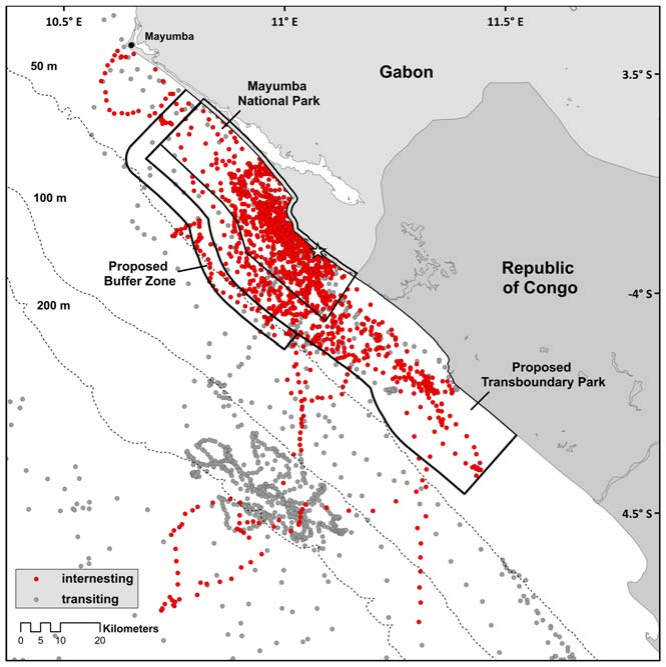
All state-space modeled tracks (n = 18) of olive ridley sea turtles (*Lepidochelys olivacea*) tagged from Mayumba National Park. Red points represent internesting behavioral mode; grey points represent transiting behavioral mode. Star indicates tagging location.

Mayumba National Park (MNP) is a 900 km^2^ marine protected area (IUCN Category II National Park) encompassing 60 km of coastline in Gabon, Africa just north of the Republic of Congo border. Two key species of conservation concern found in the park are leatherback and olive ridley sea turtles (*Lepidochelys olivacea*) [40], [41]. While only several hundred olive ridley nests are laid in the park every year, the park hosts between 5000 and 20,000 nesting leatherbacks annually [42], [43]. Strandings of olive ridley sea turtles, however, are disproportionately higher (59 to 95% of stranded turtles) than leatherback turtles in both Gabon and the Republic of Congo, with mortality largely attributed to fisheries bycatch and entanglement [44], [45]. This suggests that the park is not effectively protecting ridleys from fishing mortality. Fine-scale internesting movements by leatherback turtles in the region surrounding MNP are relatively well-understood [33] and have fostered the desire for cross-border collaboration in the form of a Transboundary Park (TBP) proposed between Gabon and the Republic of Congo (Figure 1). The TBP would expand MNP's current park boundaries, increasing the size of the protected region by over 1400 km^2^. There is, however, a paucity of data regarding the movements of olive ridleys in the region and an increased understanding may allow management and enforcement resources to be more adequately partitioned for the most effective protection of this species. Accordingly, we monitored olive ridley movements by satellite tracking to determine the internesting movements of olive ridleys in Central Africa, how these movements relate to MNP and the proposed TBP and previously determined leatherback sea turtle movements, and the effects of satellite telemetry location error on our confidence in animal movement in relation to current and proposed park boundaries. Drawing from this work, we provide a framework for effective management of breeding individuals of long-lived marine species in order to effectively use the limited resources for conservation of this and similar species.

## Methods

A Spanish translation of this article (Text S1) and a French translation of the abstract (Text S2) are available as supporting information.

### Ethics statement

The animal use protocol for this research was reviewed and approved by the University of California Santa Cruz Institutional Animal Care and Use Committee. Procedures were approved under Gabon Agence Nationale des Parcs Nationaux permit #012-PR-CNPN-PNM.

### Study area and sea turtle tracking

We studied the internesting movements of 18 female olive ridleys in the 2007-08 (n = 5) and 2008-09 (n = 13) nesting seasons at Mayumba National Park (MNP), Nyanga Province, Gabon, Africa (Figure 1, Table 1). The nesting season for olive ridleys begins in late September or early October, peaks in late November or early December and ends by February, with occasional nests recorded through June [40]. Animals were tagged early in the nesting season in order to capture as much of the internesting period as possible though we cannot be certain that the nesting event at the time of tag attachment was the first nest of the season. Tags were deployed from Base Camp Nyafessa (3.96° S, 11.15° E), the highest density location of olive ridleys nesting in MNP. Tag attachment procedure lasted approximately 40 minutes and began about 10 minutes after nesting activity was initiated in order to reduce handling time. If additional time was needed to complete the tagging process, animals were physically restrained by hand for a maximum of 30 minutes.

**Table 1:** Summary of internesting (IN) behavior.

Year	Turtle	Tag date	CCL	CCW	Total time in IN mode (days)	Approx. date(s) of renesting event(s)	Renesting interval (days)	Approx dist from tag location	Time in IN mode after last nest	Max dist north	Max dist south	Max dist off-shore
2007-08	A	15 Nov	71	75	25.4	6 Dec	21	<10 km	4.4	33	18	21
	B	14 Nov	69	71	18.5	2 Dec	18	<10 km	0.5	11	12	17
	C	13 Nov	70	70	22.9	1 Dec	18	<10 km	4.9	28	83	51
	D	05 Nov	71	71	8.8*	-	-	-	*	18*	0*	17*
	E	16 Nov	70	66	31.0	25 Nov, 16 Dec	9, 21	<10 km	1.0	17	56	97
	F	12 Nov	69	71	19.2	30 Nov	18	<60 km	1.2	53	23	21
	H	20 Nov	72	71	**	8 Dec	18	<10 km	**	30	3	12
	I	14 Nov	70	71	0	-	-	-	0.0	-	-	-
	J	14 Nov	66	67	11.3	9 Dec	25	<10 km	-	22	14	16
	K	19 Dec	70	69	25.4	5 Jan, 20 Jan	17, 15	<70 km	8.4	53	54	44
	L	04 Dec	71	72	0	-	-	-	0.0	-	-	-
	M	04 Dec	69	70	22.3	24 Dec	20	<10 km	2.3	0	71	14
2008-09	N	13 Nov	76	74	25.4*	23 Nov	10	<70 km	*	69*	8*	18*
	O	07 Nov	71	70	16.9	26 Nov	18	<10 km	1.1	35	31	22
	P	07 Nov	71	74	6.0	-	-	-	6.0	15	11	23
	Q	08 Nov	71	72	24.0*	-	-	-	*	16*	32*	33*
	R	09 Nov	71	72	6.9*	-	-	-	*	15*	24*	10*
	Mean (SD)		70.5 (2.0)	70.9 (2.3)			∼17.5		2.7 (2.8) (n = 11)	27.7 (18.6)	29.3 (25.4)	27.7 (22.3)

*Tag died before departing zone or changing behavior mode

**Remained in internesting mode for most of track; max distances were calculated using portion of the track prior to last nesting event; total time in internesting mode and prior to last nest were not calculated.

Turtle G transmitted for only 3.1 d and was excluded from further analyses. Turtles I and L departed immediately in transit mode following transmitter attachment. Abbreviations are as follows: curved carapace length (CCL), curved carapace width (CCW).

If not already present, turtle front flippers were tagged with uniquely numbered monel metal tags [46], and curved carapace length and width were recorded. Turtles were equipped with either KiwiSat 101 (n = 12, 440 g (in air), Sirtrack Ltd, Havelock North, New Zealand) or Telonics ST20, Model A1010 (n = 6, 276 g (in air), Mesa, AZ, USA) satellite platform transmitter terminals attached using Sika Anchorfix 3 epoxy (Lyndhurst NJ, USA). Animals were not weighed, however adult female olive ridley mass averages approximately 35 kg [47]; thus tags were less than 2% of adult ridley mass and the whole attachment, including resin, was close to neutrally buoyant. Data were collected via the Argos satellite system [48] and automatically downloaded and parsed via the Satellite Tracking and Analysis Tool (STAT) [49].

### Track analysis using state-space models

A behaviorally switching state-space model (SSM) was fitted to Argos tracks to handle observation error, improve data retention, and infer animal behavioral state from the movement pattern [50]. Argos location data, though an improvement over previous behavioral estimates, can be highly erroneous due to the Doppler algorithm used to calculate location during satellite overpasses [51], [52]. Not accounting for this error can have marked effects on analyses and the conclusions of movement and behavior [37], [39]. Additionally, common statistical approaches for understanding animal movement are based on assumptions of independence, such that crucial features of movement such as spatio-temporal autocorrelation are handled by discarding valuable data or handling it in ad-hoc ways [38], [53], [54]. State-space models directly address these issues by coupling a model for observation error with a mechanistic model of animal movement and solving the models together [53]. This results in better location estimates as well as estimates of the uncertainty of location estimates. To determine uncertainty, the SSM draws on the statistical power of the whole dataset as well as an animal's expected behavior as parameterized by the mechanistic model [55]. These uncertainties can then be carried into subsequent analysis so that error is properly propagated forward.

Using the free software packages R and WinBUGS, we fit the behaviorally switching SSM initially developed by Jonsen et al. [53] and refined by Breed et al. [50] to each turtle track. We estimated locations and associated credible limits at five-hour intervals; we chose this time interval as it reflects the average number of Argos locations per day for these animals. Following Bailey and colleagues [56], behavior was discriminated into two states that we nominally refer to as: “internesting” (state 1) and “transiting” (state 2). Behavioral modes were based on two parameters: mean turning angle (θ) and autocorrelation in speed and direction (γ). A lack of overlap between the parameters representing the opposing behavioral states indicated a true differentiation in movement patterns. For this analysis, only internesting portions of the track were used and the remainder of the track was discarded from further analysis.

### Characterization of internesting movements

After objectively determining the internesting portion of tracks using the SSM, internesting movements where further characterized using a number of common metrics:

- Renesting events and internesting interval: In this study, the tagging date is the only confirmed nesting event. Previous studies have used haulout loggers built into tags or factors such as increased location quality due to time on land, directed onshore movement and/or direct observation to determine renesting events [19], [26], [33], [34], [57]. Due to low Argos satellite coverage near the equator, short nesting times (approximately 45 mins) and the remote nature of the nesting beaches, we could not determine exact dates and times of renesting events. Instead, renesting events were inferred based on (a) directed nearshore movement and (b) occurrence of these movements within the average known renesting interval of olive ridley sea turtles (between 6 and 30 days [20]). Often, the renesting event could be inferred to within only a two-day range; thus, renesting dates and intervals are approximate.
- Nesting site fidelity: The straight-line distance from the original tagging location and successive inferred nests determined nesting site fidelity. As renesting events were approximate in both time and space, the exact renesting location could not be determined; thus distance from the original tagging location is reported in increments of 10 km.
- Distance and direction moved between nests: To characterize internesting movements, the maximum distance and direction (characterized for simplicity as north, south and offshore, though note that the coast of Gabon is not oriented directly north-south) from the original tagging location were calculated for each turtle, and the mean in each direction reported for the tagged population.
- Post-nesting movements: Time turtles remained in the internesting mode following the last nesting event before switching to the transiting behavior mode was determined for all turtles which transmitted through the entire internesting period (termed the ‘post-nesting’ portion of the track).

### Turtle distribution within marine protected area and political boundaries

Home range analyses were applied to characterize how olive ridleys used territorial waters of Gabon and Congo, and the existing and proposed marine protected areas. There are many home range methodologies available, each with their respective benefits and drawbacks [58], [59]. Spatial scales of analysis and research questions are important considerations in choosing a home range method [60], [61]. We chose grid cells for this application for two reasons. First, we wanted precise measurements of animal distribution given our study questions and gridding allowed us to see finer scale movements even when data were aggregated across animals. Second, the small spatial scale of the analysis resulted in over-smoothed results using methods such as kernel density estimation or convex hulls, masking movement on the scale appropriate for this study. It is important to note that grid cell size can have marked effects on study output [62], however there is no standard method of choosing grid cell size. Thus, we felt that the appropriate grid cell size should be as fine as possible to best define small-scale movements, but large enough to produce smooth contours as an individual animal moved from one grid cell to the next (i.e. reducing gaps between successively used cells). Using this reasoning, we chose a grid cell size of 32 km^2^ for successive analyses.

We determined turtle distribution within the waters of: (a) Mayumba National Park (current boundaries), (b) the proposed Transboundary Park, (c) the proposed MNP Buffer Zone, (d) the Gabonese Exclusive Economic Zone (EEZ), and (e) the Congolese EEZ. Use of these areas was characterized using a utilization distribution (UD) of the number of positions per grid cell. The UD is defined as a probability distribution of finding an animal in any given cell within a defined time frame [57]. The UD was calculated by first determining the number of positions per grid cell and then normalized to the proportion of total locations per grid cell by dividing by the total number of locations used in the analyses. These proportions were sorted from largest to smallest and the cumulative proportion of locations per grid cell were determined to create UDs. This was done using custom tools in R (Version 2.8, R Core Team) and ArcGIS (Version 9.3, ESRI). Core areas were defined as areas used most intensely, and quantified as where space use deviated the greatest from random, following Powell [63]. Core areas were subsequently defined as UDs of 80% or less.

### Confidence intervals

The error of Argos locations can be many kilometers [37], [51] and this can have marked effects on analysis outcomes, especially when analyses are conducted on small spatial scales [37], [64]. Given the scale of Mayumba National Park (900 km^2^) and the proposed Transboundary Park (approximately 2300 km^2^) and the proximity of these boundaries to internesting turtle movements, Argos error could lower our confidence that turtles remain within park boundaries, potentially displacing fishing effort to ‘unseen’ high-density areas outside of current or proposed park boundaries. Consequently, we incorporated error estimates from the SSM to determine the effect of error on analyses.

To do this, we estimated variance surrounding each location from SSM parameters using posterior distributions. The state-space model was fit using a Bayesian Markov chain Monte Carlo method that estimates posterior distributions for all locations and parameters. Depending upon the quality and number of Argos observations, posterior distributions of location estimates were wider (when there were fewer, poorer quality Argos observations) or narrower (when there were more, higher quality Argos observations). From the posterior distributions of location estimates, the SSM yields variance (standard deviations and 95% credible limits) for each location with narrower posterior distributions resulting in smaller variance.

Variance was estimated for both latitude and longitude because Argos error varies between latitudinal and longitudinal components [50]. We assumed a normal distribution surrounded the error of the latitudinal and longitudinal components of each point. Using the standard deviation for each component to define the normal distribution, we resampled 100 points for each latitudinal and longitudinal point. The number of locations per grid cell for the 80 and 100 percent utilization distributions were then calculated (as above) for the resampled points (herein referred to as the ‘resampled SSM’ tracks) and compared to the 80 and 100 percent utilization distributions of the SSM output used in the analyses above (herein referred to as the ‘mean SSM’ tracks). Difference in area for the resampled and mean SSM analyses were calculated to give a confidence interval of high-use area.

## Results

### State-space model outputs and general track characteristics

The general movement pattern of tagged animals was to remain in the vicinity of MNP until shortly after the last nest, followed by a departure south to presumed feeding grounds off Angola. Of the 18 animals tagged, two animals (Turtles I and L) switched to transiting mode and departed the region within 24 hours of the tagging event. One tag (Turtle G) transmitted for only 3.1 days with poor quality locations; this animal was excluded from subsequent analyses. Four tags (Turtles D, N, Q and R) ceased transmitting before the animals switched from internesting to transiting mode.

Tracks showed strong separation between the behavioral parameters (θ and γ). Turtle E switched to the transiting behavioral mode for approximately 10 hours, then switched back to the internesting behavioral mode for two days and likely renested. Because the animal was nearshore, remained in the internesting habitat, and later shifted back to the transiting behavioral mode followed by typical southward movement, we included both internesting mode portions of the track and the brief transiting behavioral mode locations (total of two locations) in the analysis. Additionally, Turtle H remained in internesting mode for the entire four months she was tracked. She did move offshore (approximately 55 km) of Mayumba NP after approximately one month, likely to forage given the length of time and behavior displayed in the offshore region. As there was no clear behavioral shift, we chose to truncate her track for the internesting analysis using the boundary of the Gabonese Contiguous Zone.

### Characterization of internesting movements

- Renesting events and internesting interval: Thirteen renesting events were inferred from eleven turtles (i.e. two turtles renested twice) (Table 1). The average time between nests was approximately 17.5 days.
- Nesting site fidelity: Of the thirteen renesting events, eight were less than 10 km from the tagging site, one was less than 60 km from the tagging site, and two were less than 70 km from the tagging site (Table 1).
- Distance and direction between nests: Movements surrounding the original tagging location were relatively symmetrical in all directions (Table 1). Turtles moved an average of 27.7 km north (range = 0–53, SD = 18.6), 29.3 km south (range = 0–56, SD = 25.4), and 27.7 km offshore (range = 10–51, SD = 22.3).
- Post-nesting movements: Of the nine turtles with full internesting tracks, average time in the internesting mode before shifting to the transiting behavior mode and departing for foraging grounds was 2.7 days (SD = 2.8) (Table 1).

### Turtle distribution within marine protected area and political boundaries

**Figure 2:**
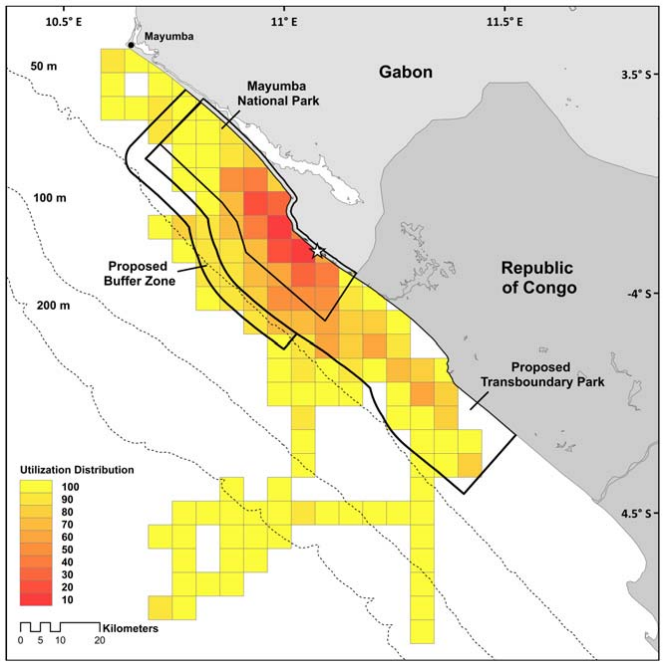
Utilization distribution (UD) of olive ridley sea turtles (*Lepidochelys olivacea*) tagged from Mayumba National Park. The UD shows that the proposed Transboundary Park encompasses the majority of the turtle distribution. Star indicates tagging location.

High density regions were found closer to the original nesting location and were well encompassed by the boundaries of the Transboundary Park, though less so by the current boundaries of Mayumba National Park (Table 2, Figure 2). MNP encompassed only 44.6% (565.3 km^2^) of the 80% UD while the proposed transboundary park encompassed almost the entire 80% UD (97.6%, 1237.3 km^2^) (Figure 2, Table 2). A similar pattern was seen for the 100% UD. The proposed buffer zone encompassed 3.7% (47.0 km^2^) of the 80% UD but the buffer zone was important in the overall distribution (69.0% of the buffer zone was used by turtles at some point). The Gabonese EEZ encompassed more of the 80% UD (66.7%, 845.6 km^2^) than the Congolese EEZ, however, overall the Congolese EEZ was used more than the Gabonese EEZ (Congolese EEZ: 53.7% or 2369.0 km^2^ of the 100% UD).

**Table 2:** Use of marine protected areas and political zones by olive ridley sea turtles during the internesting period.

	Zone (total area, km^2^)	MNP(969.0)	TBP (2818.0)	Buffer Zone only(419.8)	Gabonese waters*	Congolese waters*
80% (total area = 1267.7 km^2^)	km^2^	565.3	1237.2	47.0	845.6	423.5
	% total IN track	44.6	97.6	3.7	66.7	33.4
100% UD (total area = 4414.8 km^2^)	km^2^	841.4	2387.0	289.7	2048.2	2369.0
	% total IN track	19.1	54.1	6.6	46.4	53.7

*The exact boundary between Gabon and Congo is unclear, resulting in some overlap between the calculated zones.

Abbreviations are as follows: Mayumba National Park (MNP), proposed Transboundary Park (TBP).

### Confidence intervals

**Figure 3:**
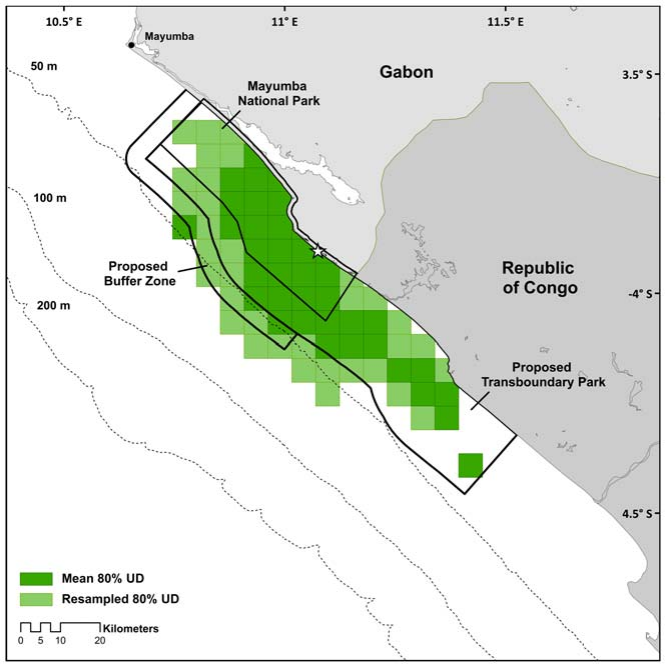
Confidence intervals of movements for olive ridley sea turtles (*Lepidochelys olivacea*) tagged from Mayumba National Park. Outer error bounds for 80% utilization distribution for mean SSM estimates (light green) and resampled SSM estimates (dark green) are shown. Star indicates tagging location.

The high-use area (80% UD) of the resampled tracks showed a similar pattern to that of mean SSM tracks in that the majority of the high-use area was concentrated in the proposed TBP (88.0% resampled vs. 97.6% mean SSM) and only a third of high-use regions occurred within MNP (Table 3, Figure 3). The mean SSM tracks showed 3.7% of high use area in the proposed buffer zone, but when error was incorporated, the amount of high-use area in the buffer zone almost tripled (to 9.7%) within this small region. Not surprisingly, the 100% UD showed greater variability between the resampled tracks and mean SSM tracks than the 80% UD. The total area of the 100% UD for the resampled tracks was 4.6 times greater than the mean.

**Table 3:** Summary of marine protected area usage between mean and resampled state-space modeled tracks using utilization distributions (UD).

		Total Area	% total UD in TBP	% total UD in MNP	MNP	TBP	Buffer Zone only	Gabonese EEZ	Congolese EEZ
**80% UD: No. hits per cell (km^2^)**	Mean SSM	1267.7	97.6	44.6	565.3	1237.2	47.0	845.6	423.5
	Resampled SSM	2368.8	88.0	33.0	781.9	2084.9	229.4	1523.3	846.0
	% difference	46.5	9.6	11.6	27.7	40.7	79.5	44.5	49.9
**100% UD: No. hits per cell (km^2^)**	Mean SSM	4414.8	54.1	19.1	841.4	2387.0	289.7	2048.2	2369.0
	Resampled SSM	20376.9	13.8	4.4	906.7	2818.0	419.8	7077.3	13299.6
	% difference	78.3	40.2	14.6	7.2	15.3	31.0	71.1	82.2

Abbreviations are as follows: Mayumba National Park (MNP), proposed Transboundary Park (TBP).

## Discussion

### Conservation implications of internesting movements

**Figure 4:**
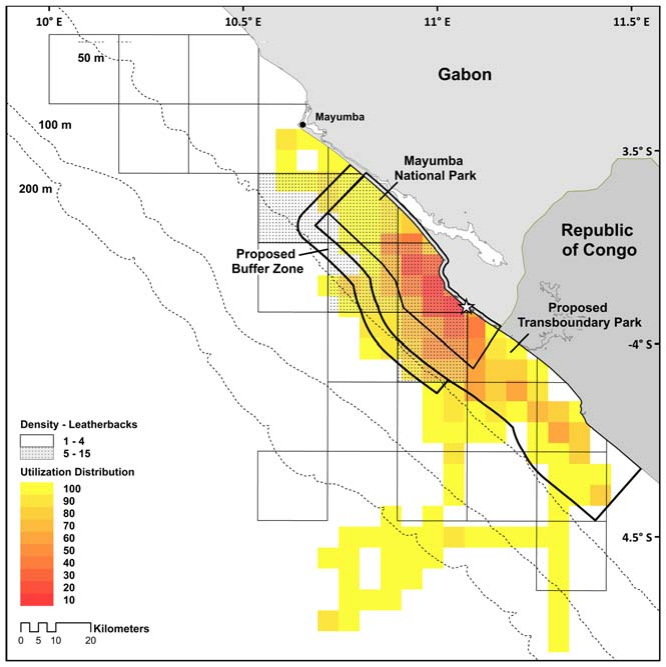
Density of leatherback sea turtles (*Dermochelys coriacea*) (from [**33**]) overlaid with olive ridley (*Lepidochelys olivacea*) utilization distribution, showing similar distributions and effectiveness of park boundaries. Star indicates tagging location.

Effective conservation of species of concern occurs when the appropriate scale, life history stage and opportunities converge [4], [27], [29], [30]. Gabon and the Republic of Congo are working to enact conservation strategies within their borders, despite limited resources to do so, and results of this study create a picture of tractable conservation for the nesting olive ridley population of Central Africa. When olive ridley distributions are further overlaid by leatherback sea turtle distributions determined in a previous study (Figure 4, [33]), we see that both turtle species are confined to the same region, highlighting the multi-species importance of this area. Analyses of internesting movements revealed that females remain confined to a small region (∼30 km radius) centered around the original tagging location, and usually returned to within 10 km of the original tagging site in subsequent nesting attempts, corroborating results of research on solitary olive ridley nesters in Northern Australia [65], [66], French Guiana [24], Surinam [47] and Costa Rica [67]. Individual movements were generally focused along shore in shallow waters (less than 50 m, Figure 1), creating a focused zone for protection such as found for loggerhead sea turtles in Greece [68] and green turtles at Ascension Island [69]. Thus, protection for nesting females may be confined both spatially and temporally, and the limited movements of females from this high-density nesting site increases the importance of protecting the internesting grounds [70]. Variations in this movement trend may result from individual variation, similar to that seen in the foraging strategies in other turtle and large pelagic marine species [57], [71]–[74], such as leatherback turtles in French Guiana whose internesting dispersal radius varied by over 100 km in the same season [75].

### State-space models: Management and study design implications

The inferred behavioral state from the SSM highlights the importance of considering turtle behavior after the final nesting event in management strategies. Similar to leatherback sea turtles [56], olive ridleys remained in the internesting mode for approximately three days following their final nesting event (Table 1). Though tracks were not state-space modeled, Hamel et al [65] also noted that two nesting olive ridley turtles tagged off Northern Australia remained near to the nest site for three to four days after the last nest was laid. In sea turtle literature, the nesting season is commonly defined to end after the last nesting event, however our results suggest that when larger-scale behavior is taken into account, the nesting season may extend for several days after the last nest, perhaps in order to recover from physically taxing reproductive activities before departing on long-distance migrations to foraging grounds [76]. This suggests that animals may be exposed to the suite of nearshore anthropogenic threats for an additional period after the last nest is laid, and may be in a somewhat physically compromised state during that period. Similarly, both male and female turtles are exposed to these threats during the mating period prior to when the first nest is laid. This further highlights the need for comprehensive protection of turtle nesting grounds, as turtles are concentrated in a discrete area for long periods of time [17].

SSMs additionally aided in defining conservation needs. SSMs have been repeatedly shown to improve track quality and data retention [53], [55], [56], and others have used state-space models or comparisons between Argos and GPS data to show the uncertainty in conclusions about animal movement when raw Argos data is used [37], [77]–[80]. We used the Bayesian credible limits estimated by the SSM to (1) gauge our level of confidence in space-use estimates and (2) incorporate uncertainty into management recommendations. Given the fine spatial scale of this study, error from Argos locations could have had marked effects on our confidence in how animals are moving in relation to MPA boundaries. For example, though the proposed buffer zone incorporates only 3.7% of high-density use areas using the mean SSM tracks, error estimates show that the buffer zone may be incorporating more high-density use areas and that these areas may extend further offshore (Figure 3). This highlights the importance of the buffer zone in the face of uncertainty and leads us to strongly recommend for the inclusion of the buffer zone in the TBP, and for considering expansion of its boundaries further offshore and south. Through this analysis, our level of confidence in turtle distribution can be incorporated into future management plans by planning park boundaries and enforcement strategies using a precautionary management approach.

Geographic Positioning System (GPS) tags have become an important technological advancement over Argos in tracking studies [18], [37], however, we further highlight the benefits of combined use of Argos data and state-space models when GPS studies are not possible. While GPS data ideally results in more accurate locations, there are financial and logistical constraints associated with using GPS tags. In order to track sea turtles by GPS without recapturing animals, data must still be uploaded by the Argos system. This results in satellite time costs, as well as the cost of tags that house GPS capabilities. These tags are currently three to four times more expensive than the most inexpensive Argos tag. In order to track animals by GPS without incurring additional satellite costs, animals must be recaptured which is difficult with sea turtles given their infrequent contact with land, and is even more difficult in remote regions. Additionally, recent analyses have shown that despite the quality of data received from GPS, Argos data is as accurate as GPS data if Argos tags provide data at regularly spaced intervals [81]. Combining Argos data and state-space models improves track quality and data retention, and also provides robust measures of derived behavior. As technology improves and costs are lowered in tandem, GPS tracking for sea turtles and other marine animals will undoubtedly become more feasible but for now, Argos tracking may represent the most cost effective option in many scenarios [37], particularly when used in conjunction with data processing techniques such as state-space modeling [81].

### Regional protection of sea turtles

Internesting movements in relation to the current and proposed MPA boundaries in this region showed that management strategies in this region are on target to provide comprehensive protection to the nesting populations of both olive ridley and leatherback sea turtles. Mayumba National Park encompasses a large percentage of high use areas for turtles (44.6%), but does not adequately protect all high-use regions (Figure 2). By contrast, extending protection to include the proposed Transboundary Park will incorporate 97.6% of high-use areas, as well as incorporate 84.7% of the total area used by turtles in this study. Furthermore, the creation of the TBP is required to protect Gabonese nesting turtles that spend more than half (53.7%) of their time in Congolese waters, with similar patterns shown for leatherback turtles (Figure 4, [19], [33]), highlighting the need for international protection. Our study additionally suggests the need for nearshore protection for olive ridleys in other areas along the West African coast that are currently underprotected and the need to better understand internesting distributions of olive ridleys along the entire African coast. Protection in Gabon and in other regions must be implemented at an international level to effectively conserve this species in Africa.

### Conclusions: Satellite Tracking as a Conservation Tool

This study considerably advances our understanding of not only the internesting movements and behavior of Atlantic olive ridley populations, but also those of solitary nesting olive ridleys that, thus far, have received little scientific study [82], [83]. We see a clear pattern of turtles in this population remaining nearshore and close to the tagging nesting site throughout the internesting range. This pattern highlights a clear opportunity for viable conservation measures for female nesting olive ridleys in Central Africa, and potentially in other non-*arribada* nesting sites around the world, though we recommend further study at Congolese and other Central African nesting sites to verify this pattern in other regional populations.

Through this project we additionally define a framework for conservation of breeding individuals of long-lived species using satellite telemetry as a primary tool. First, we initiated a project with a clear, spatially driven management question. We selected a region with a high density of an imperiled species but tractable conservation opportunities given the existence of a marine reserve and managers motivated to reduce bycatch. With this in place we designed a short-term project that would inform long-term management goals. Short-term projects can provide vital information for refining how existing but limited funds can be more effectively used over the long term. The biological and life history information collected by our focused telemetry project allows for better enforcement and park structure, and the information has direct long-term sustainability and conservation applications. Third, we explicitly considered the limitations of our methodologies (satellite tracking) in conjunction with management strategies. In many instances, understanding the spatial scale of analyses and drawbacks to methods used is critical to streamline management and enforcement for better conservation outcomes, but these caveats are rarely considered. Through this study, we provide a structure for adaptive management and suggest that despite the inherent difficulties in protecting far-ranging pelagic animals, there exist distinct and impactful opportunities for conserving long-lived species.

## Supporting Information

Text S1 **Spanish translation of the article.**
(PDF)

Text S2 **French translation of the abstract.**
(PDF)
