# Supplementary material for: Using Satellite Tracking to Optimize Protection of Long-Lived Marine Species: Olive Ridley Sea Turtle Conservation in Central Africa
Source: PLoS One. 2011 May 11;6(5):e19905. doi: 10.1371/journal.pone.0019905 (PMC3092776; doi:10.1371/journal.pone.0019905)
Supplement: Text S1 — Spanish translation of the article. (PDF) [file pone.0019905.s001.pdf]

Uso de Telemetría Satelital para Optimizar la Protección de Especies Marinas de Vida Larga: Conservación de la Tortuga Golfina en Africa Central

Sara M. Maxwell[1*‡], Greg A. Breed[2], Barry A. Nickel[3], Junior Makanga-Bahouna[4], Edgard Pemo-Makaya[4], Richard J. Parnell[4], Angela Formia[5], Solange Ngouessono[6], Brendan J. Godley[7], Daniel P. Costa[2], Matthew J. Witt[7], Michael S. Coyne[7,8]

[1]Ocean Sciences Department, University of California Santa Cruz, Santa Cruz CA 95060 USA
[2]Department of Ecology and Evolutionary Biology, University of California Santa Cruz, Santa Cruz CA 95060 USA
[3]Center for Integrated Spatial Research, University of California Santa Cruz, Santa Cruz CA 95060 USA
[4]Wildlife Conservation Society, Parc National de Mayumba, Mayumba, Gabon
[5]Wildlife Conservation Society, Global Conservation Program, New York NY 10460 USA
[6]Agence Nationale des Parcs Nationaux, Libreville, Gabon
[7]Marine Turtle Research Group, Centre for Ecology and Conservation, University of Exeter – Cornwall Campus, Penyrn, Cornwall, TR10 9EZ UK
[8]SEATURTLE.org, Durham NC 27705, USA
*Corresponding author: smaxwell@ucsc.edu , +1 206 355 3249
[‡]Current address: Marine Conservation Institute, Long Marine Lab, 100 Shaffer Road, Santa Cruz CA 95060 USA

Palabras clave: Tortuga marina golfina; *Lepidochelys olivacea*; áreas marinas protegidas; conservación internacional; modelo de estado espacial; rango habitacional; telemetría satelital; error en movimiento animal

RESUMEN

El monitoreo de las medidas de conservación para especies de vida larga, requiere de la interacción entre la protección de estadíos de historia de vida relevantes en el sentido biológico y de condiciones que sean socio-económicamente factibles. La protección de los adultos reproductivos requiere del conocimiento de los patrones de movimiento de los animales, y de cómo estos movimientos se relacionan con los límites políticos, así como también requiere de cierto nivel de confianza en los análisis de movimientos espaciales. Utilizamos telemetría satelital y un modelo de estado especial para determinar los movimientos entre-desove de la tortuga marina golfina (*Lepidochelys olivacea*) (n = 18) en Africa Central durante dos temporadas de desove (2007-08, 2008-09). Estos movimientos fueron analizados en relación a los límites actuales del parque, así como en relación a los límites de un parque transnacional propuesto entre Gabón y la República del Congo, ambos creados con el fin de reducir la captura incidental de tortugas marinas en pesquerías marinas. Adicionalmente, determinamos los intervalos de confianza para las estimaciones de los rangos habitacionales. Inicialmente, las tortugas permanecieron dentro de un radio de 30 km del sitio de desove, para luego abandonar la zona hacia áreas de alimentación más distantes. Solo el 44.6 por ciento de

las áreas de alta densidad se ubicaron dentro de los límites actuales del parque, pero el parque transnacional propuesto incorpora el 97.6 por ciento éstas. Aunque originalmente los animales rastreados satelitalmente provinieron de Gabón, las tortugas frecuentaron aguas congolesas durante más de la mitad del período entre-desoves (53.7 por ciento), destacando la necesidad de cooperación internacional y de proveer apoyo científico para el parque transnacional propuesto. Este es el primer estudio comprehensivo de los movimientos de tortugas golfinas reproductivas solitarias, y sugiere la oportunidad de establecer medidas de manejo para el monitoreo de hembras reproductivas de tortugas golfinas en éste y otros sitios de desove en el mundo. Nuestros resultados nos permiten proponer un marco conceptual para la protección de especies de vida larga, utilizando telemetría satelital como herramienta principal.

Introducción

La protección de los recursos naturales es una prioridad global, sin embargo la implementación de medidas de conservación en un contexto socio-político complejo constituye un desafío importante. [1-3]. La implementación de medidas de conservación tangibles para animales de vida larga requiere que la protección de estadíos de vida relevantes sea logística, política y económicamente factible [4-6]. Aunque estudios han demostrado la vulnerabilidad de estadíos de vida tempranos para algunas especies marinas (e.g. tortugas marinas [7,8], aves marinas [9], elasmobranquios [10], pinipedios [11]), la protección de adultos reproductivos de especies de vida larga logra sustentar a la población a través de dos mecanismos. Primero, los animales reproductivos contribuyen, de manera desproporcionada, a sustentar la población en comparación con individuos no reproductivos [6,12]. Segundo, para muchas especies, las actividades reproductivas ocurren en regiones geográficas particulares y durante varios meses. Sin embargo, a pesar de que dichas regiones discretas son, frecuentemente, altamente vulnerables, éstas permiten la protección práctica de la especie en comparación a otros casos donde los individuos se encuentran más dispersos [13,14].

Las tortugas marinas constituyen excelentes candidatos, así como también modelos, para la protección de áreas reproductivas discretas y vulnerables. Las temporadas de desove o anidación de las tortugas marinas generalmente se extienden por varios meses, período durante el cual las hembras regresan repetidamente a la misma playa para desovar [15]. A pesar de que varias especies son altamente migratorias en otras partes de su rango distribucional, tanto las hembras como los machos de las tortugas marinas regresan desde zonas de alimentación distantes, y permanecen en la vecindad de las playas de anidación para el apareamiento y desove o anidación, por lo que los individuos reproductivos se agregan espacio- y temporalmente [16-20]. Ya que tanto los machos como las hembras ocupan áreas similares debido a las actividades de apareamiento y reproducción, es altamente probable que la protección del espacio ocupado por las hembras incluya también la distribución de los machos [17]. Sin embargo, la protección adecuada de las hembras requiere del conocimiento de tres aspectos claves: (1) los movimientos de los animales entre eventos de desove; (2) saber cómo estos movimientos se relacionan con el manejo de la especies y los límites políticos; y (3) nuestro nivel de confianza en la precisión de las inferencias de

movimientos, dados los métodos utilizados en los análisis de las escalas espaciales implicadas.

Los movimientos entre-desove varían considerablemente entre especies, y la comprensión de estos movimientos es crítica para su protección efectiva. Algunas tortugas bobas o caguama (*Caretta caretta*) permanecen dentro de un radio de unos pocos kilómetros del lugar original de desove, mientras que tanto la tortuga laúd (*Dermochelys coriacea*) como la tortuga verde (*Chelonia mydas*) pueden cubrir cientos de kilómetros entre lugares de desove [21-25]. Así, el conocimiento de las escalas espacio-temporales de los movimientos entre-desove determina las escalas en las que son necesarias las medidas de protección, ayudando así en la implementación de medidas de manejo en un contexto apropiado a las necesidades humanas y ecológicas. La telemetría satelital ha demostrado ser una herramienta efectiva en la obtención de conocimiento acerca del comportamiento de los animales en el mar, permitiendo determinar los movimientos de los animales alejados de la tierra, siendo especialmente útil en playas de anidación remotas donde las tortugas no son fácilmente recapturadas [26].

El segundo elemento a considerar para la protección exitosa, es entender cómo estos movimientos se relacionan con las estrategias de manejo espaciales, como por ejemplo las Areas Marinas Protegidas (AMPs). Las AMPs son ampliamente utilizadas en la protección de especies sensibles, reduciendo actividades como la pesca dentro de sus límites, pero son solo efectivas en la medida que los límites sean establecidos adecuadamente, incorporando todas las áreas de importancia utilizadas por la especie, y solo si los límites de dichas áreas son respetados [27-31]. Si las AMPs son diseñadas sin un conocimiento certero de las distribuciones de la(s) especie(s) a proteger, se puede derivar en un desplazamiento no intencional, concentrando el esfuerzo de pesca en áreas no protegidas que, sin embargo, corresponden a áreas de alta utilización por la especie que se pretende conservar [32].

La telemetría satelital ha probado ser un medio efectivo para observar cómo la biología y el comportamiento animal se relacionan con los límites políticos [25,33-36], pero esto conlleva al tercer elemento crítico en la protección adecuada: nuestro nivel de confianza en los datos de distribución espacial, dadas las limitaciones conocidas de dichas metodologías. El error inherente asociado con datos de telemetría satelital puede reducir la confianza en nuestras estimaciones de posiciones y densidades. Cuando el área bajo observación corresponde a una escala espacial menor al error de la estimación satelital, los análisis e inferencias pueden verse negativamente impactados [37-39] lo que podría resultar en la definición equívoca de los límites de protección de medidas como las AMPs. Sin embargo, nuevos avances en el procesamiento de los datos de telemetría satelital nos permiten considerar el error de observación de forma robusta. Los modelos de estado espacial logran separar el error de observación de los procesos de comportamiento en los análisis del movimiento de los animales. Esto permite a los investigadores la estimación de intervalos de confianza en cada ubicación, y obtener mejores estimaciones de los parámetros biológicos [38]. Dichos intervalos de confianza pueden ser usados para informar análisis espaciales posteriores, permitiéndonos considerar este error al recomendar medidas de conservación tales como la posición de

los límites de un parque.

El Parque Nacional Mayumba (PNM) corresponde a un área marina protegida de 900 km^2 (UICN, Parque Nacional Categoría II), que abarca 60 km de la costa de Gabón, Africa, al norte de la frontera con la República del Congo. Dos especies de gran importancia en los esfuerzos de conservación se encuentran en el parque: la tortuga laúd y la tortuga golfina (*Lepidochelys olivacea*) [40,41]. Si bien el número de nidos de las tortugas golfinas en el parque es sólo del orden de algunos cientos, el parque concentra entre 5 000 y 20 000 tortugas laúd en anidación anualmente [42,43].

Sin embargo, los números de tortugas golfinas varadas son desproporcionadamente mayores (59 al 95% de los varamientos) a los varamientos de tortugas laúd, tanto en Gabón como en la República del Congo, con mortalidades ampliamente atribuidas a las capturas incidentales en pesquerías y enmalles [44,45]. Lo anterior sugiere que el parque no está protegiendo a las tortugas golfina de mortalidades en la pesquería de manera efectiva. Los movimientos entre-desove de pequeña escala de las tortugas laúd alrededor del PNM son bien conocidos [33] y han estimulado los deseos de colaboración transnacionales en la forma de un Parque Transnacional (PTN), propuestos entre Gabón y la República del Congo (Figura 1). El PTN expandiría los límites actuales del PNM, incrementando el tamaño de la región protegida en más de 1 400 km^2. Sin embargo, existe una carencia de datos respecto de los movimientos de las tortugas golfinas en la región, a pesar de que un mejor entendimiento podría ayudar en su manejo, permitiendo que los recursos sean dirigidos, de forma más adecuada, hacia medidas de protección más efectivas para la especie. Nosotros seguimos los movimientos de las tortugas golfinas usando telemetría satelital, para poder así determinar los movimientos entre-desove de dicha especie en África Central, la relación de estos movimientos con el PNM y el PTN propuesto, así como con los movimientos de las tortugas laúd (previamente determinados), y los efectos del error en las ubicaciones obtenidas por telemetría satelital en nuestra confianza al determinar los movimientos de la especie en relación a los límites actuales del parque y los propuestos. En base a los resultados obtenidos en este trabajo, presentamos un marco para el manejo efectivo de individuos reproductivos de especies marinas de vida larga, con el fin de usar efectivamente los limitados recursos disponibles para la conservación de ésta y otras especies similares.

Métodos

Ética animal

El protocolo de uso animal para este estudio fue revisado y aprobado por el Comité Institucional de Cuidado y Uso Animal (CICUA) de la Universidad de California Santa Cruz. Todos los procedimientos fueron aprobados bajo el permiso #012-PR-CNPN-PNM de la Agencia Nacional de Parques Nacionales de Gabón.

Área de estudio y seguimiento de tortugas marinas

Estudiamos el comportamiento entre-desove de 18 hembras de tortuga golfina durante las temporadas de desove 2007-08 (n = 5) y 2008-09 (n = 13), en el Parque Nacional Mayumba (PNM), Provincia de Nyanga, Gabón, África (Figura 1, Tabla 1). La temporada de desove de la Tortuga golfina empieza a fines de Septiembre o principios de Octubre,

llegando a un máximo a fines de Noviembre o principios de Diciembre, para finalizar en Febrero, registrándose desoves ocasionales hasta el mes de Junio [40]. Las tortugas fueron capturadas a principios de la temporada de desove para lograr cubrir la mayor parte del período entre-desoves, aunque no hay certeza de que el desove al momento de captura fuera el primero de la temporada. Las capturas se realizaron en la Base Campamento Nyafessa (3.96° S, 11.15° E), donde se registra la más alta densidad de tortugas golfinas desovando en el PNM. La manipulación de los animales, que se extendió por aproximadamente 40 minutos, comenzó alrededor de 10 minutos después de iniciado el desove, para así minimizar el tiempo de manipulación de los animales. En aquellos procedimientos que se extendieron para completar la adhesión de los instrumentos, los animales fueron manualmente controlados por un período no superior a los 30 minutos.

Los individuos fueron marcados con bandas metálicas monel con números únicos [46], en aquellos casos en que no estuvieran presentes, y la longitud y ancho del caparazón fueron registrados. Las tortugas fueron equipadas con marcas satelitales KiwiSat 101 (n=12, 440 g (en aire), Sirtrack Ltd, Havelock North, Nueva Zelanda), o Telonics ST20, Modelo A1010 (n=6, 276 g (en aire), Mesa, AZ, EE.UU), adheridas usando pegamento epóxico Sika Anchorfix 3 (Lyndhurst NJ, EE.UU). Los animales no fueron pesados, sin embargo hembras adultas de la especie tortuga golfina promedian cerca de 35 kg [47]; por lo que las marcas satelitales correspondieron a menos del 2% de la masa de ejemplares hembras adultas, y el montaje completo, incluyendo la resina, es casi neutralmente boyante. Los datos fueron recolectados vía el sistema satelital Argos [48] y obtenidos automáticamente y procesados por medio de la Herramienta para el Rastreo Satelital y Análisis (Satellite Tracking and Analysis Tool, STAT) [49].

Análisis de movimientos usando Modelos de Estado Espacial

Un modelo de estado espacial (MEE) fue ajustado a los datos de posición Argos para poder determinar el error de observación, mejorar la retención de datos e inferir el estado comportamental a partir del patrón de movimiento [50]. A pesar de constituir un mejoramiento en las estimaciones comportamentales de animales, los datos de ubicación de Argos pueden contener errores importantes debido al algoritmo Doppler utilizado para calcular la posición durante el paso del satélite [51,52]. El ignorar este error puede tener efectos importantes en los análisis y conclusiones obtenidas de datos de movimiento y comportamiento [37,39]. Por otro lado, métodos estadísticos de uso común en estudios de movimiento animal están basados en supuestos de independencia, lo que implica que características propias del movimiento, como la auto-correlación espacio-temporal, sean tratadas removiendo datos, o con manipulaciones ad-hoc [38,53,54]. Los modelos de estado espacial (MEE) abordan directamente dichos aspectos, al acoplar un modelo para la observación del error con un modelo mecanicista del movimiento animal, y solucionando ambos modelos en conjunto [53]. Lo anterior resulta en una mejor estimación de los datos de ubicación, así como estimaciones de la incerteza de los mismos. Para determinar dicha incerteza, el MEE se basa en el poder estadístico del set de datos completo, así como del comportamiento esperado del animal, parametrizado por el modelo mecanicista [55]. Estas incertezas pueden ser

acarreadas en análisis subsecuentes, de forma tal que el error es propagado en los pasos posteriores.

Usando los paquetes computacionales gratuitos R y WinBUGS, ajustamos el modelo de cambio de estado especial, inicialmente desarrollado por Jonsen et al. [53] y posteriormente refinado por Breed et al. [50] para cada individuo. Estimamos los datos de posición, a los que asociamos límites factibles en intervalos de cinco horas; dicho intervalo fue seleccionado ya que refleja el número promedio diario de ubicaciones Argos animales. Siguiendo lo propuesto por Bailey y colegas [56], el comportamiento fue discriminado en dos estados, a saber: "entre-desove" (estado 1) y "tránsito" (estado 2). Los modelos comportamentales se basaron en dos parámetros: ángulo promedio de giro (θ) y auto-correlación en velocidad y dirección (γ). La falta de solapamiento entre los parámetros, representando estados comportamentales opuestos, indica la diferenciación verdadera en el patrón de movimiento. Para este análisis, únicamente las porciones entre-desove fueron usadas, y el resto del registro fue descartado del resto de los análisis.

Caracterización de los movimientos entre-desove

Luego de determinar objetivamente la porción entre-desove de los datos usando el MEE, los movimientos entre-desove fueron caracterizados en detalle usando un número de parámetros de uso común:

(a) Eventos de desove e intervalos entre-desove: En este estudio, la fecha de captura constituye el único evento de desove confirmado. Estudios anteriores han utilizado sensores de tiempo en tierra incorporados en los instrumentos, u otros factores como el aumento en la calidad de los datos de ubicación debido al tiempo en tierra, movimientos dirigidos hacia tierra y/u observación directa para determinar eventos posteriores de desove [19,26,34,42,57]. Debido a la pobre cobertura de los satélites Argos cerca del Ecuador, los cortos períodos de desove (aproximadamente 45 min) y la naturaleza remota de las playas de desove, no pudimos determinas las fechas o tiempos exactos de los eventos de desove. Por ello, los eventos de desove fueron inferidos en base a (a) movimientos dirigidos a tierra, y (b) presencia de dichos movimientos dentro del intervalo de desove descrito para la especie (entre 6 y 30 días [20]). A menudo, los eventos de desove solo pudieron ser determinados en el rango de uno a dos días; por lo que las fechas de desove e intervalos son aproximados.

(b) Fidelidad al sitio de desove: La distancia en línea recta entre la ubicación de captura original y los sitios de desove sucesivos inferidos determinan la fidelidad al sitio de desove. Ya que los eventos de desove fueron aproximados tanto en tiempo como en espacio, la ubicación exacta del sitio de desove no pudo ser determinada; por lo que la distancia del sitio original de captura es reportada en incrementos de 10 km.

(c) Distancia y dirección abarcada entre desoves: Para caracterizar los movimientos entre-desove, la distancia máxima y la dirección (caracterizada, por conveniencia como norte, sur y mar adentro, a pesar de que la costa de Gabón no está orientada en dirección norte-sur) desde la ubicación original de captura para cada tortuga, y el promedio en cada dirección es reportado para la población estudiada.

(d) Movimientos post-desove: El tiempo que las tortugas permanecieron en el modo entre-desove, luego del último evento de desove y previo al cambio al comportamiento de tránsito, fue determinado para todas las tortugas que transmitieron durante el período entre-desove completo (denominado porción 'post-desove')

Distribución de las tortugas dentro del Area Marina Protegida y los Límites Políticos
Análisis del rango habitacional fue empleado para caracterizar cómo las tortugas golfina usaron las aguas territoriales de Gabón, Congo, y del Area Marina Protegida existente y propuesta. Existen diversas metodologías para analizar los rangos habitacionales, cada cual con sus respectivas fortalezas y debilidades [58,59]. Las escalas espaciales de los análisis y de las preguntas a investigar son aspectos clave al elegir un método para el análisis de rango habitacional [60,61]. En nuestro estudio, elegimos una grilla por dos razones. Primero, queríamos contar con mediciones precisas de la distribución de las tortugas dadas las preguntas de nuestro estudio, y el uso de una grilla nos permite ver movimientos en escalas más finas, aún cuando los datos estén agregados entre individuos. Segundo, lo reducido de la escala espacial de nuestros análisis derivó en resultados sobre-ajustados al utilizar métodos como densidades de kernel o cascos convexos, enmascarando el movimiento en la escala apropiada para este estudio. Es importante mencionar que, a pesar que el tamaño de las celdas de la grilla puede tener un efecto importante en los resultados del estudio [62], no existe un método estándar para elegir el tamaño de las celdas. Basado en lo anterior, decidimos que el tamaño de las celdas de la grilla debieran ser lo más pequeño posible para poder definir movimientos de pequeña escala, pero lo suficientemente grande como para producir contornos suavizados cuando un animal se desplaza de una celda de la grilla a la siguiente (i.e. reduciendo el espacio entre las celdas sucesivamente utilizadas). Usando este razonamiento, elegimos un tamaño de celda de 32 km^2 para nuestros análisis.

Determinamos la distribución de las tortugas dentro de las aguas de: (a) el Parque Nacional Mayumba (límites actuales), (b) el Parque Transnacional propuesto, (c) la zona de amortiguación propuesta para el PNM, (d) la Zona Económica Exclusiva (ZEE) gabonesa, y (e) la ZEE congolesa. El uso de estas aguas fue caracterizado usando la Distribución de Utilización (DU) del número de posiciones por celda. La DU se define como la distribución de la probabilidad de encontrar un animal en una celda determinada en un tiempo definido [57]. La DU fue calculada primero determinando el número de posiciones por celda y luego normalizando la proporción of posiciones totales por celda, dividiéndola por el número total de posiciones usadas en los análisis. Estas proporciones fueron ordenadas de mayor a menor, y la proporción acumulativa de posiciones por celda fue determinada para crear las DUs. Esto fue hecho usando herramientas desarrolladas en R (Versión 2.8, R Core Team) y ArcGIS (Versión 9.3, ESRI). Las áreas núcleo fueron definidas como las áreas utilizadas más intensamente, y cuantificadas como las zonas donde el uso del espacio de desvió mayormente de un proceso aleatorio, siguiendo a Powell [63]. Las áreas núcleo fueron luego definidas como las DUs de 80% o menos.

Intervalos de Confianza

El error de las posiciones Argos puede ser de varios kilómetros [37,51] y esto puede tener efectos importantes en los resultados, especialmente cuando los análisis son llevados a cabo en escalas espaciales pequeñas [37,64]. Dadas las escalas del Parque Nacional Mayumba (900 km^2) y del Parque Transnacional propuesto (aproximadamente 2300 km^2) y la cercanía de los límites de éstos con los movimientos entre-desove de las tortugas, el error de Argos puede disminuir nuestra confianza de que las tortugas permanecen dentro de los límites de los parques, potencialmente desplazando el esfuerzo de pesca hacia zonas de alta densidad 'no detectadas' fuera de los límites actuales o propuestos. Consecuentemente, incorporamos estimaciones de error del MEE para determinar el efecto del error en los análisis.

Para ellos, estimamos la varianza alrededor de cada posición usando las distribuciones posteriores. El Modelo de Estado Espacial fue ajustado usando el método Bayesiano de Cadenas Markov Monte Carlo, que estima distribuciones posteriores para todas las posiciones y parámetros. Dependiendo de la calidad y número de posiciones Argos, las distribuciones posteriores de las posiciones estimadas fueron más amplias (cuando hubo menos posiciones Argos y de menor calidad), o más estrechas (cuando hubo más observaciones Argos y de mejor calidad). A partir de las distribuciones posteriores de las posiciones estimadas, el MEE provee la varianza (desviación estándar y 95% límites de credibilidad) para cada posición con distribuciones posteriores más estrechas, resultando en una varianza menor.

La varianza fue estimada tanto para la latitud como para la longitud, ya que el error de Argos varía entre los componentes latitudinales y longitudinales [46,47]. Nosotros asumimos una distribución normal alrededor del error de ambos componentes para cada punto. Usando la desviación estándar para cada componente, para luego definir la distribución normal, re-muestreamos 100 puntos para cada punto latitudinal y longitudinal. El número de posiciones por celda de la grilla para las Distribuciones de Utilización de 80 y 100% fueron calculadas (como se explica anteriormente) para los puntos re-muestreados (de aquí en adelante denominados como 'posiciones re-muestreadas del MEE') y comparadas con las Distribuciones de Utilización de 80 y 100% de los resultados del MEE usados en los análisis anteriores (de aquí en adelante denominados como 'posiciones promedio del MEE'). La diferencia de área entre las posiciones re-muestreadas y promedio del MEE fueron calculadas para obtener un intervalo de confianza de áreas de uso importante.

Resultados

Resultados del Modelo de Estado Espacial y características generales de los movimientos

El patrón general de movimiento de los animales en este estudio fue permanecer en las cercanías del PNM hasta poco después del último desove, para luego partir hacia el sur, probablemente en busca de zonas de alimentación en Angola. De los 18 animales capturados, dos individuos (tortugas I y L) cambiaron a modo de tránsito y abandonaron la región a menos de 24 horas de la captura. Un individuo (tortuga G) transmitió por solo 3.1 días, con posiciones de baja calidad; este animal no fue incluido en los análisis posteriores. Cuatro individuos (tortugas D, N, Q y R) cesaron de transmitir antes que los

animales cambiaran de modo entre-desove a modo tránsito.

Los resultados mostraron una fuerte separación entre los parámetros comportamentales (θ y γ). La tortuga E cambió al modo comportamental de tránsito por cerca de 10 horas, para luego volver al modo comportamental entre-desove por dos días y posiblemente volvió a desovar. Debido a que el animal se encontraba cerca de la costa, permaneció en el hábitat de desove, y cambió luego al modo comportamental de tránsito seguido por un desplazamiento típico hacia el sur, decidimos incluir tanto la porción entre-desove de su registro, como las posiciones durante su breve modo comportamental de tránsito (total de dos posiciones) en el análisis. Adicionalmente, la tortuga H permaneció en modo entre-desove por los 4 meses que duró su registro. Esta hembra si se aventuró mar adentro (aproximadamente 55 km) del PNM luego de alrededor de un mes, posiblemente para alimentarse dada la duración de esta fase así como el comportamiento observado en dicha región más oceánica. Ya que no hubo un cambio comportamental claro, decidimos acortar su registro usando solo la fase entre-desove dentro de los límites de la Zona Contigua gabonesa.

Caracterización de los movimientos entre-desove

(a) Eventos de desove e intervalos entre-desoves: Trece eventos de desove fueron inferidos para once tortugas (i.e. dos tortugas desovaron dos veces) (Tabla 1). El tiempo promedio entre desoves fue de aproximadamente 17.5 días.
(b) Fidelidad al sitio de desove: De los trece eventos de desove, ocho fueron a menos de 10 km del sitio de captura, y otros dos fueron a menos de 70 km del sitio de captura (Tabla 1).
(c) Distancia y dirección entre desoves: Los movimientos alrededor del sitio de captura fueron relativamente simétricos en todas las direcciones (Tabla 1). Las tortugas se desplazaron en promedio 27.7 km al norte (rango=0-53, DE=18.6), 29.3 km al sur (rango=0-56, DE=25.4), y 27.7 km mar adentro (rango=10-51, DE=22.3).
(d) Movimientos post-desove: De las nueve tortugas con registros entre-desove completos, el tiempo promedio en modo entre-desove antes de cambiar al modo de tránsito y desplazarse a zonas de alimentación, fue de 2.7 días (DE=2.8) (Tabla 1).

Distribución de las tortugas dentro del Area Marina Protegida y Límites Políticos

Las zonas de alta densidad fueron identificadas cerca de la ubicación del desove original y estuvieron contenidas dentro de los límites del Parque Transnacional, y en menor nivel dentro de los límites del Parque Nacional Mayumba (Tabla 2, Figura 2). El PNM abarcó solo el 44.6% (565.3 km^2) de la DU del 80%, mientras que el Parque Transnacional propuesto abarcó casi la totalidad de la DU del 80% (97.6%, 1237.3 km^2) (Figura 2, Tabla 2). Un patrón similar fue observado para la DU del 100%. La zona de amortiguación abarcó el 3.7% (47.0 km^2) de la DU del 80%, pero esta zona fue de mayor importancia en la distribución general (69.0% de la zona de amortiguación fue utilizadas por las tortugas en algún momento). La ZEE gabonesa abarcó más de la DU del 80% (66.7%, 845.6 km^2) que la ZEE congolesa, sin embargo, en términos generales la ZEE congolesa fue usada más que la ZEE gabonesa (ZEE congolesa: 53.7% o 2369.0 km^2 de la DU del 100%).

Intervalos de Confianza
Las áreas de alta utilización (DU del 80%) de las posiciones re-muestreadas mostraron un patrón similar al de las posiciones promedio del MEE, ambas mostrando que la mayoría de las áreas de alta utilización estuvieron concentradas en el PTN propuesto (88.0% posiciones re-muestreadas vs. 97.6% posiciones promedio MEE) y solo un tercio de las áreas de alta utilización estuvieron dentro del PNM (Tabla 3, Figura 3). Las posiciones promedio MEE mostraron un 3.7% de las áreas de alta utilización en la zona de amortiguación propuesta, pero cuando se incorporó el error, las áreas de alta utilización en la zona de amortiguación casi se triplicaron (hasta 9.7%) dentro de este pequeña región. Con ello, no es sorprendente que la DU del 100% mostró mayor variabilidad entre las posiciones re-muestreadas y las posiciones promedio MEE que la DU del 80%. El área total de la DU del 100% para las posiciones re-muestreadas fue 4.6 veces mayor que para las posiciones promedio.

Discusión
La conservación efectiva de especies se logra hacer coincidir la escala espacial, el estadío de vida y las oportunidades apropiadas [4,27,29,30]. Gabón y la República del Congo están trabajando en conjunto para implementar estrategias de conservación dentro de sus límites, a pesar de las limitaciones en los recursos para dicha tarea, y los resultados de este estudio crean un escenario de conservación monitoreable para la población reproductiva de la tortuga golfina en Africa Central. Si se sobrepone la distribución de la tortuga golfina con la distribución de la tortuga laúd, determinada en estudios previos (Figura 4, [33]), vemos que ambas especies de tortugas se encuentran restringidas a la misma región, destacando la importancia de esta zona para diversas especies. El análisis de los movimientos entre-desove reveló que las hembras permanecieron confinadas en una región pequeña (radio ~30 km) alrededor de la ubicación de captura original, y usualmente regresaron a una distancia de menos de 10 km de la misma en intentos posteriores de desove, corroborando los resultados de investigaciones de tortugas golfina solitarias en el norte de Australia [65,66], Guyana Francesa [24], Surinam [47] y Costa Rica [67]. Los movimientos individuales generalmente se concentraron a lo largo de la costa en aguas someras (menos de 50 m, Figura 1), creando una zona específica para concentrar la protección como la hallada para tortugas caguama (o bobas) en Grecia [68] y tortugas verde en la Isla Ascensión [69]. Así, la protección de hembras desovantes puede confinarse tanto espacial, como temporalmente, y lo limitado de los movimientos de las hembras en este sitio de alta densidad de desoves aumenta la importancia de proteger estos sitios de desove [70]. Las variaciones en esta tendencia de movimiento puede ser el resultado de variaciones individuales, similar a lo observado en estrategias de alimentación en otras especies de tortugas u otras especies pelágicas de gran tamaño [57,71-74], como las tortugas laúd en la Guyana Francesa, cuyos radios entre ubicaciones de desove varían en más de 100 km durante la misma temporada [75].

El estado comportamental inferido del MEE destaca la importancia de considerar el comportamiento de las tortugas luego del último evento de desove en las estrategias de manejo. Como ocurre con las tortugas laúd [56], las tortugas golfina permanecieron

en modo entre-desove por cerca de tres días luego de su último desove (Tabla 1). Aunque sus resultados no provienen de un MEE, Hamel et al [65] también encontraron que dos tortugas golfinas en desove, capturadas en el norte de Australia, permanecieron cerca del sitio de captura por tres o cuatro días después del último desove. En la literatura científica, el fin de la temporada de desove se define comúnmente como el último evento de desove, sin embargo nuestros resultados sugieren que, cuando se considera el comportamiento de gran escala, la temporada de desove puede extenderse por varios días después del último evento de desove, posiblemente para recuperarse de lo demandante de las actividades reproductivas antes de partir en migraciones de largo alcance hacia las zonas de alimentación [76]. Esto sugiere que los animales pueden estar expuestos a una serie de amenazas antropogénicas cerca de la costa por un lapso adicional de tiempo luego del último desove, pudiendo además estar físicamente comprometidos durante ese período. Asimismo, tanto los machos como las hembras se ven expuestos a dichas amenazas durante el período reproductivo, previo al primer desove. Esto supone la importancia de una protección comprehensiva de los sitios de desove de las tortugas, ya que éstas se concentran en un área por períodos de tiempo prolongados [17].

Los MEEs ayudaron a definir las necesidades de conservación. En repetidas ocasiones, se ha demostrado que los MEEs mejoran la calidad de los datos de posiciones, y mejoran la retención de los datos [53,55,56], y otros estudios comparando datos Argos con posiciones GPS muestran la incerteza en las conclusiones a las que se llega cuando se utilizan los datos Argos crudos [37,77-80]. Nosotros usamos los límites de credibilidad Bayesianos estimados por el MEE para (1) ajustar nuestro nivel de confianza en las estimaciones de utilización del espacio, y (2) incorporar la incerteza en recomendaciones de manejo. Dada lo fino de la escala espacial de este estudio, el error de las posiciones Argos pudo haber tenido efectos importantes en nuestra confianza en cómo los animales se mueven en relación a los límites de la AMP. Por ejemplo, aunque la zona de amortiguación propuesta solo incorpora el 3.7% de las áreas de alta utilización usando las posiciones promedio del MEE, estimaciones de error muestran que dicha zona de amortiguación puede incorporar más áreas de alta utilización, las cuales se pueden extender mar adentro (Figura 3). Esto resalta la importancia de la zona de amortiguación al considerar la incerteza, y nos hace recomendar la inclusión de la zona de amortiguación en el PTN, así como la expansión de los límites del mismo hacia el sur y mar adentro. Por medio de este análisis, nuestro nivel de confianza en la distribución de las tortugas puede ser incorporado en planes de manejo futuros al planificar los límites del parque e implementando estrategias usando un enfoque de manejo preventivo.

Instrumentos de rastreo animal con Sistemas de Posicionamiento Geográfico (GPS por sus siglas en inglés) constituyen un avance tecnológico con respecto a Argos [18,37], sin embargo, creemos necesario resaltar los beneficios del uso combinado de datos Argos con modelos de estado espacial cuando estudios con GPS no son posibles. Si bien los datos de GPS idealmente proveen resultados con posiciones más certeras, existen limitaciones financieras y logísticas asociadas con los instrumentos GPS. Para poder estudias tortugas marinas con GPS sin recapturar a los animales, los datos aún

tienen que ser enviados vía el sistema de satélites Argos. Ello conlleva costos por el uso de los satélites, así como el costo del instrumento con capacidad GPS. Estos últimos tienen un costo de entre tres y cuatro veces el costo de un instrumento Argos. Para poder seguir animales con GPS sin incurrir en costos adicionales por uso de los satélites, los animales tienen que ser recapturados, lo que es difícil con tortugas marinas, dado la poca frecuencia con que visitan la tierra, and es aún más difícil en regiones remotas. Asimismo, análisis recientes han demostrado que, a pesar de la calidad de los datos recibidos de instrumentos GPS, los datos Argos llegan a ser tan precisos como los datos GPS si los instrumentos Argos proveen datos en intervalos de tiempo regulares [81]. El combinar datos Argos con modelos de estado espacial mejora la calidad de los datos de posiciones, así como la retención de datos, y provee medidas robustas del comportamiento derivado. Sin duda que, con el avance de la tecnología y la disminución en los costos, datos de GPS para tortugas marinas y otras especies marinas se harán más comunes, pero por ahora, las posiciones Argos pueden representar la opción más específica desde un punto de vista de costo-beneficio, en diversos escenarios [37], en particular cuando son usados junto con técnicas de procesamiento como los modelos de estado espacial [81].

Los movimientos de desove de las tortugas en relación con los límites de la AMP actuales y propuestos en esta región demuestran que las estrategias de manejo en esta zona están bien dirigidas hacia ofrecer una protección comprehensiva de las poblaciones desovantes de las tortugas golfina y laúd. El Parque Nacional Mayumba abarca un gran porcentaje de las áreas de alta utilización de las tortugas (44.6%), pero no protege todas las áreas de alta utilización de forma adecuada (Figura 2). En contraste, el extender la zona protegida para incluir el Parque Transnacional propuesto, incorporará el 97.6% de las áreas de alta utilización, así como incorporará el 84.7% del área total usada por las tortugas en este estudio. Aún más, se requiere de la creación del PTN para proteger las tortugas desovantes de Gabón, las cuales pasan más de la mitad de su tiempo (53.7%) en aguas congolesas, con patrones similares a los observados para las tortugas laúd (Figura 4, [19,33]), destacando la necesidad de contar con protección internacional. Nuestro trabajo sugiere, además, la necesidad de protección costera para la tortuga golfina en otras áreas de la costa de Africa Noroccidental, que se encuentran actualmente desprotegidas, así como la necesidad de mejorar nuestro conocimiento de la distribución de las tortugas golfina a lo largo de la costa del continente africano. La protección en Gabón y en otras regiones debe ser implementada a un nivel internacional para poder conservar, efectivamente, a esta especie en el continente.

Conclusiones

Este estudio constituye una contribución considerable para nuestro conocimiento de los movimientos entre-desove y el comportamiento, no solamente de la población de tortugas golfina del Atlántico, sino que también de aquellos individuos reproductivos solitarios que, hasta ahora, han sido pobremente estudiados [82,83]. Observamos un patrón claro en que las tortugas en esta población permanecen cerca de la costa y cerca al lugar de captura a lo largo del rango espacial de desove. Este patrón constituye una

oportunidad clara para implementar medidas de conservación viables para las hembras reproductivas de tortuga golfina en Africa central, y potencialmente en otros sitios de desove en el mundo. Sin embargo, recomendados expandir los estudios en sitios de anidación en la República del Congo y otros sitios de desove en Africa central para poder así verificar este patrón en otras poblaciones locales.

A través de este proyecto logramos definir un marco para la conservación de individuos reproductivos de especies de vida larga usando telemetría satelital como herramienta principal. En primera instancia, iniciamos un proyecto con una pregunta clara, dirigida hacia el manejo espacial de la especies. Logramos seleccionar una región con altas densidades de una especie en peligro, donde las oportunidades de conservación, considerando la existencia de una reserva marina, han logrado motivar a los entes involucrados en el manejo a reducir la captura incidental. Con ellos, diseñamos un proyecto a corta escala que nos proveería información para los objetivos de manejo a gran escala. Proyectos a corta escala pueden contribuir información vital para refinar cómo los limitados recursos existentes pueden ser usados más efectivamente en el largo plazo. La información biológica y de la historia de vida recolectada por nuestro proyecto de telemetría, permite la mejor concentración de los esfuerzos y recursos del parque, y la información es sustentable en el largo plazo y tiene aplicaciones en la conservación. En tercer lugar, explícitamente consideramos las limitaciones de nuestras metodologías (rastreo satelital) en el contexto de las estrategias de manejo. En diversas instancias, el entender las escalas espaciales de los análisis y las limitaciones de los métodos empelados es crítico para mejorar el manejo y asegurar mejores resultados de las medidas de conservación, pero dichas limitaciones raramente son consideradas. Con este estudio, proveemos una estructura para el manejo adaptativo y sugerimos que, a pesar de las dificultades inherentes al proteger animales pelágicos de amplia distribución, existen oportunidades claras y de claro impacto para conservar especies de vida larga.

Agradecimientos
Agredecemos a A. Mavoungou Djimbi Alain, S. Zassi, E. Ibouanga Amos, L. Schonkneckt y M. Markovina por su apoyo en el campo, SEATURTLE.org, UCSC Center for Integrated Spatial Research, H. Bailey y S. Bograd por su asistencia invaluable, Gabon Sea Turtle Partnership por el apoyo para el monitoreo de los desoves y el personal de Wildlife Conservation Society, en particular Q. Makaya Pambou, por el apoyo logístico. Gracias a S. Fossette, G. Hays y C. Champagne por sus comentarios en el manuscrito y L. Huckstadt por traducción. SMM desea agradecer K. Jenkins, D. Wingfield, C. Champagne, L. Crowder, C. y J. Square, A. Sanders, L. Keith, A. Whittaker, S. Bograd, M. Cole, W. Goodman y TWIG por el apoyo durante este proyecto.

Referencias

1. Root TL, Schneider SH (2006) Conservation and climate change: The challenges ahead. Conservation Biology 20: 706-708.
2. Ehrenfeld D (2000) War and peace and conservation biology. Conservation Biology 14: 105-112.
3. Sarkar S, Pressey RL, Faith DP, Margules CR, Fuller T, et al. (2006) Biodiversity conservation planning tools: Present status and challenges for the future. Annual Review of Environment and Resources 31: 123-159.
4. Godley BJ, Barbosa C, Bruford M, Broderick AC, Catry P, et al. (2010) Unravelling migratory connectivity in marine turtles using multiple methods. Journal of Applied Ecology 47: 769-778.
5. Carwardine J, Wilson KA, Watts M, Etter A, Klein C, et al. (2008) Avoiding Costly Conservation Mistakes: The Importance of Defining Actions and Costs in Spatial Priority Setting. PLoS ONE 3: e2586.
6. Gerber LR, Heppell SS (2004) The use of demographic sensitivity analysis in marine species conservation planning. Biological Conservation 120: 121-128.
7. Pritchard PCH (1980) The Conservation of Sea Turtles - Practices and Problems. American Zoologist 20: 609-617.
8. Crouse D, Crowder L, Caswell H (1987) A stage-based population model for loggerhead sea turtles and implications for conservation. Ecology 68: 1412-1423.
9. Finkelstein ME, Doak DF, Nakagawa M, Sievert PR, Klavitter J (2010) Assessment of demographic risk factors and management priorities: impacts on juveniles substantially affect population viability of a long-lived seabird. Animal Conservation 13: 148-156.
10. Frisk MG, Miller TJ, Fogarty MJ (2002) The population dynamics of little skate *Leucoraja erinacea*, winter skate *Leucoraja ocellata*, and barndoor skate *Dipturus laevis*: predicting exploitation limits using matrix analyses. ICES Journal of Marine Science 59: 576-586.
11. Craig MP, Ragen TJ (1999) Body size, survival, and decline of juvenile Hawaiian monk seals, *Monachus schauinslandi*. Marine Mammal Science 15: 786-809.
12. Heppell SS, Crowder LB, Menzel TR (1999) Life table analysis of long-lived marine species with implications for conservation and management. In: Musick J, editor. Life in the Slow Lane: Ecology and Conservation of Long-Lived Marine Animals. Bethesda MD: American Fisheries Society. pp. 137-146.
13. Danchin E, Wagner RH (1997) The evolution of coloniality: the emergence of new perspectives. Trends in Ecology & Evolution 12: 342-347.
14. Meylan A (1995) Behavioral ecology of the West Caribbean green turtle (*Chelonia mydas*) in the internesting habitat. In: Bjorndal K, editor. Biology and Conservation of Sea Turtles, Revised Edition. Washington DC: Smithsonian Institution Press. pp. 67-80.
15. Vanbuskirk J, Crowder LB (1994) Life-History Variation in Marine Turtles. Copeia: 66-81.
16. Plotkin P, Owens D, Byles R, Patterson R (1996) Departure of male olive ridley turtles (*Lepidochelys olivacea*) from a nearshore breeding ground. Herpetologica 52: 1-

7.
17. Hays GC, Fossette S, Katselidis KA, Schofield G, Gravenor MB (2010) Breeding Periodicity for Male Sea Turtles, Operational Sex Ratios, and Implications in the Face of Climate Change. Conservation Biology 24: 1636-1643.
18. Schofield G, Bishop CM, MacLean G, Brown P, Baker M, et al. (2007) Novel GPS tracking of sea turtles as a tool for conservation management. Journal of Experimental Marine Biology and Ecology 347: 58-68.
19. Georges J-Y, Fossette S, Billes A, Ferraroli S, Fretey J, et al. (2007) Meta-analysis of movements in Atlantic leatherback turtles during the nesting season: conservation implications. Marine Ecology Progress Series 338: 225-232.
20. Miller J (1997) Reproduction in Sea Turtles. In: Lutz P, Musick J, editors. The Biology of Sea Turtles. Boca Raton FL: CDC Press. Pp 51-81.
21. Schofield G, Hobson VJ, Lilley MKS, Katselidis KA, Bishop CM, et al. (2010) Inter-annual variability in the home range of breeding turtles: Implications for current and future conservation management. Biological Conservation 143: 722-730.
22. Blumenthal J, Solomon J, Bell C, Austin T, Ebanks-Petrie G, et al. (2006) Satellite tracking highlights the need for international cooperation in marine turtle management. Endangered Species Research 7: 1-11.
23. Eckert KL, Eckert SA, Adams TW, Tucker AD (1989) Inter-Nesting Migrations by Leatherback Sea Turtles (*Dermochelys coriacea*) in the West-Indies. Herpetologica 45: 190-194.
24. Georges J-Y, Fossette S, Laur M, Martini S, Plot V, et al. (2007) At sea movements and diving behavior of olive ridley turtles during and after the nesting season in French Guiana: conservation implications. In: Rees A, Frick M, Panagopoulou A, Williams K, editors; Myrtle Beach SC. NOAA pp. 43.
25. Shillinger G, Swithenbank A, Bograd S, Bailey H, Castelton M, et al. (2010) Identification of high-use internesting habitats for eastern Pacific leatherback turtles: role of the environment and implications for conservation. Endangered Species Research 10: 215-232.
26. Tucker AD (2010) Nest site fidelity and clutch frequency of loggerhead turtles are better elucidated by satellite telemetry than by nocturnal tagging efforts: Implications for stock estimation. Journal of Experimental Marine Biology and Ecology 383: 48-55.
27. Pollnac R, Christie P, Cinner J, T D, Daw T, et al. (2010) Marine reserves as linked social-ecological systems. Proceedings of the National Academy of Sciences 107: 18262-18265.
28. Costello C, Rassweiler A, Siegel D, De Leo G, Micheli F, et al. (2010) The value of spatial information in MPA network design. Proceedings of the National Academy of Sciences 107: 18294-18299.
29. Roberts C (2000) Selecting marine reserve locations: Optimality versus opportunism. Bulletin of Marine Science 66: 581-592.
30. Agardy T (2000) Information needs for marine protected areas: Scientific and societal. Bulletin of Marine Science 66: 875-888.
31. Agardy T, di Sciara GN, Christie P (2011) Mind the gap: Addressing the shortcomings

of marine protected areas through large scale marine spatial planning. Marine Policy 35: 226-232.

32. Baum J, Myers R, Kehler D, Worm B, Harley S, et al. (2003) Collapse and conservation of shark populations in the Northwest Atlantic. Science 299: 389-392.
33. Witt MJ, Broderick AC, Coyne MS, Formia A, Ngouessono S, et al. (2008) Satellite tracking highlights difficulties in the design of effective protected areas for Critically Endangered leatherback turtles Dermochelys coriacea during the inter-nesting period. Oryx 42: 296-300.
34. Zbinden J, Aebischer A, Margaritoulis D, Arlettaz R (2007) Insights into the management of sea turtle internesting area through satellite telemetry. Biological Conservation 137: 157-162.
35. Hyrenbach KD, Keiper C, Allen SG, Ainley DG, Anderson DJ (2006) Use of marine sanctuaries by far-ranging predators: commuting flights to the California Current System by breeding Hawaiian albatrosses. Fisheries Oceanography 15: 95-103.
36. Wienecke B, Robertson G (2002) Foraging areas of king penguins from Macquarie Island in relation to a marine protected area. Environmental Management 29: 662-672.
37. Witt M, Akesson S, Broderick A, Coyne M, Ellick J, et al. (2010) Assessing accuracy and utility of satellite tracking data using Argos-linked Fastloc-GPS. Animal Behaviour 80: 571-581.
38. Patterson T, Thomas L, Wilcox C, Ovaskainen O, Matthiopoulos J (2008) State–space models of individual animal movement. Trends in Ecology & Evolution 23: 87-94.
39. Bradshaw C, Sims D, Hays G (2007) Measurement error causes scale-dependent threshold erosion of biological signals in animal movement data. Ecological Applications 17: 628-638.
40. Godgenger M-C, Breheret N, Bal G, N'Damite K, Girard A, et al. (2009) Nesting estimation and analysis of threats for Critically Endangered leatherback *Dermochelys coriacea* and Endangered olive ridley *Lepidochelys olivacea* marine turtles nesting in Congo. Oryx 43: 556-563.
41. Fretey J (2001) Biogeography and Conservation of Marine Turtles of the Atlantic Coast of Africa/Biogeographie et conservation des tortues marines de la cotê Atlantique de l'Afrique. Bonn, Germany: UNEP/CMS Secretariat. 254 p.
42. Witt MJ, Baert B, Broderick AC, Formia A, Fretey J, et al. (2009) Aerial surveying of the world's largest leatherback turtle rookery: A more effective methodology for large-scale monitoring. Biological Conservation 142: 1719-1727.
43. Mayumba National Park (unpublished data).
44. Van Leeuwe H, Bitsindou A (2008) Marine turtle season 2007-2008: Conkouati-Douli National Park, Republic of Congo, Final Report. Wildlife Conservation Society. 13 p.
45. Parnell R, Verhage B, Deem SJ, Van Leeuwe T, Nishihara T, et al. (2007) Marine Turtle Mortality in Southern Gabon and Northern Congo. Marine Turtle Newsletter 116: 12-14.
46. Balazs G (1999) Factors to consider in the tagging of sea turtles. In: Eckert K, Bjorndal K, Abreu-Grobois F, Donnelly M, editors. Research and Management Techniques

for the Conservation of Sea Turtles: IUCN/SSC Marine Turtle Specialist Group, Publication No. 4.
47. Schulz J (1975) Sea turtles nesting in Surinam. Zoologische Verandelingen (Leiden) 143: 3-172.
48. Argos (2008) User's Manuel. Toulouse: CLS/Service Argos.
49. Coyne M, Godley B (2005) Satellite Tracking and Analysis Tool (STAT): an integrated system for archiving, analyzing and mapping animal tracking data. Marine Ecology Progress Series 301: 1-7.
50. Breed GA, Jonsen ID, Myers RA, Bowen WD, Leonard ML (2009) Sex-specific, seasonal foraging tactics of adult grey seals (*Halichoerus grypus*) revealed by state-space analysis. Ecology 90: 3209-3221.
51. Costa DP, Robinson PW, Arnould JPY, Harrison A-L, Simmons SE, et al. (2010) Accuracy of ARGOS Locations of Pinnipeds at-Sea Estimated Using Fastloc GPS. PLoS ONE 5: e8677.
52. Vincent C, McConnell B, Ridoux V, Fedak M (2002) Assessment of Argos location accuracy from satellite tags deployed on captive gray seals. Marine Mammal Science 18: 156-166.
53. Jonsen I, Flenming J, Myers R (2005) Robust state-space modeling of animal movement data. Ecology 86: 2874-2880.
54. Austin D, McMillan JI, Bowen WD (2003) A three-stage algorithm for filtering erroneous Argos satellite locations. Marine Mammal Science 19: 371-383.
55. Jonsen ID, Myers RA, Flemming JM (2003) Meta-analysis of animal movement using state-space models. Ecology 84: 3055-3063.
56. Bailey H, Shillinger G, Palacios D, Bograd S, Spotila J, et al. (2008) Identifying and comparing phases of movement by leatherback turtles using state-space models. Journal of Experimental Marine Biology and Ecology 356: 128-135.
57. Rees A, Saady S, Broderick A, Coyne M, Papathanasopoulou N, et al. (2010) Behavioural polymorphism in one of the world's largest populations of loggerhead sea turtles, *Caretta caretta*. Marine Ecology Progress Series 418: 201-212.
58. Getz WM, Fortmann-Roe S, Cross PC, Lyons AJ, Ryan SJ, et al. (2007) LoCoH: Nonparameteric Kernel Methods for Constructing Home Ranges and Utilization Distributions. PLoS ONE 2: e207.
59. Kernohan B, Gitzen RA, Millspaugh J (2001) Analysis of Animal Space Use and Movements. In: Millspaugh J, Marzluff J, editors. Radio Tracking and Animal Populations. San Diego: Academic Press. pp. 126-168.
60. Walker J, Balling R, Briggs J, Katti M, Warren P, et al. (2008) Birds of a feather: interpolating distribution patterns of urban birds. Computers, Environment and Urban Systems 32: 19-28.
61. Nelson TA, Boots B (2008) Detecting spatial hot spots in landscape ecology. Ecography 31: 556-566.
62. Kenward R (1987) Wildlife Radio Tagging: Equipment, Field Techniques and Data Analysis. London: Academic Press. 222 p.
63. Powell R (2000) Animal home ranges and territories and home range estimators. In:

Boitani L, Fuller T, editors. Research techniques in animal ecology: controversies and consequences. New York: Columbia University Press. pp. 442.
64. Moser BW, Garton EO (2007) Effects of telemetry location error on space-use estimates using a fixed-kernel density estimator. Journal of Wildlife Management 71: 2421-2426.
65. Hamel MA, McMahon CR, Bradshaw CJA (2008) Flexible inter-nesting behaviour of generalist olive ridley turtles in Australia. Journal of Experimental Marine Biology and Ecology 359: 47-54.
66. Whiting S, Long J, Coyne M (2007) Migration routes and foraging behaviour of olive ridley turtles *Lepidochelys olivacea* in northern Australia. Endangered Species Research 3: 1-9.
67. Kalb H (1999) Behavior and physiology of solitary and arribada nesting olive ridley sea turtles (*Lepidochelys olivacea*) during the internesting period. College Station: Texas A&M University.
68. Schofield G, Bishop CM, Katselidis KA, Dimopoulos P, Pantis JD, et al. (2009) Microhabitat selection by sea turtles in a dynamic thermal marine environment. Journal of Animal Ecology 78: 14-21.
69. Hays G, Luschi P, Papi F, Del Seppia C, Marsh R (1999) Changes in behaviour during the inter-nesting period and post-nesting migration for Ascension Island green turtles. Marine Ecology Progress Series 189: 263-273.
70. Hamann M, Godfrey M, Seminoff J, Arthur K, Barata PCR, et al. (2010) Global research priorities for sea turtles: informing management and conservation in the 21st century. Endangered Species Research 11: 245-269.
71. Phillips RA, Wakefield ED, Croxall JP, Fukuda A, Higuchi H (2009) Albatross foraging behaviour: no evidence for dual foraging, and limited support for anticipatory regulation of provisioning at South Georgia. Marine Ecology-Progress Series 391: 279-292.
72. Villegas-Amtmann S, Costa D, Tremblay Y, Salazar S, Aurioles-Gamboa D (2008) Multiple foraging strategies in a marine apex predator, the Galapagos sea lion *Zalophus wollebaeki*. Marine Ecology Progress Series 363: 299-309.
73. Seminoff J, Zárate P, Coyne M, Foley D, Parker D, et al. (2008) Post-nesting migrations of Galápagos green turtles *Chelonia mydas* in relation to oceanographic conditions: integrating satellite telemetry with remotely sensed ocean data. Endangered Species Research 4: 57-72.
74. Weise MJ, Harvey JT, Costa DP (2010) The role of body size in individual-based foraging strategies of a top marine predator. Ecology 91: 1004-1015.
75. Fossette S, Ferraroli S, Tanaka H, Ropert-Coudert Y, Arai N, et al. (2007) Dispersal and dive patterns in gravid leatherback turtles during the nesting season in French Guiana. Marine Ecology Progress Series 338: 233-247.
76. Hays GC, Broderick AC, Glen F, Godley BJ, Nichols WJ (2001) The movements and submergence behaviour of male green turtles at Ascension Island. Marine Biology 139: 395-399.
77. Tremblay Y, Robinson PW, Costa DP (2009) A Parsimonious Approach to Modeling Animal Movement Data. PLoS ONE 4: e4711.

78. Gurarie E, Andrews RD, Laidre KL (2009) A novel method for identifying behavioural changes in animal movement data. Ecology Letters 12: 395-408.
79. Eckert SA, Moore JE, Dunn DC, van Buiten RS, Eckert KL, et al. (2008) Modeling loggerhead turtle movement in the Mediterranean: Importance of body size and oceanography. Ecological Applications 18: 290-308.
80. Sims DW, Righton D, Pitchford JW (2007) Minimizing errors in identifying Levy flight behaviour of organisms. Journal of Animal Ecology 76: 222-229.
81. Breed GA, Costa DP, Goebel ME, Robinson PW (2011) Electronic tracking tag programming is critical to data collection for behavioral time-series analysis. Ecosphere 2: 1-12.
82. Godley B, Blumenthal J, Broderick A, Coyne M, Godfrey M, et al. (2008) Satellite tracking of sea turtles: Where have we been and where do we go next? Endangered Species Research 4: 3-22.
83. Bernardo J, Plotkin P (2007) An evolutionary perspective on the *arribada* phenomenon and reproductive behavioral polymorphism of olive ridley sea turtles (*Lepidochelys olivacea*). In: Plotkin P, editor. Biology and Conservation of Ridley Sea Turtles. Baltimore MD: The Johns Hopkins University Press. pp. 59-88.

Tabla 1. Resumen del comportamiento entre-desove (ED). La tortuga G transmitió por solo 3.1 d y fue excluida de los análisis. Las tortugas I y L abandonaron el sitio inmediatamente luego de la adhesión del instrumento. Las abreviaciones empleadas son: longitud curva del caparazón (LCC), ancho curco del caparazón (ACC)

**Año**	**Tortuga**	**Fecha captura**	**LCC**	**ACC**	**Tiempo total en modo ED (días)**	**Fecha(s) approx. date(s) de eventos de desove**	**Intervalo entre desoves (días)**	**Dist approx del sitio de captura**	**Tiempo en modo ED luego del desove**	**Dist max norte**	**Dist max sur**	**Dist max mar adentro**
**2007-08**	**A**	15 Nov	71	75	25.4	6 Dic	21	< 10 km	4.4	33	18	21
	**B**	14 Nov	69	71	18.5	2 Dic	18	< 10 km	0.5	11	12	17
	**C**	13 Nov	70	70	22.9	1 Dic	18	< 10 km	4.9	28	83	51
	**D**	05 Nov	71	71	8.8*	-	-	-	*	18*	0*	17*
	**E**	16 Nov	70	66	31.0	25 Nov, 16 Dic	9, 21	< 10 km	1.0	17	56	97
	**F**	12 Nov	69	71	19.2	30 Nov	18	< 60 km	1.2	53	23	21
	**H**	20 Nov	72	71	**	8 Dic	18	< 10 km	**	30	3	12
	**I**	14 Nov	70	71	0	-	-	-	0.0	-	-	-
	**J**	14 Nov	66	67	11.3	9 Dic	25	< 10 km	-	22	14	16
	**K**	19 Dic	70	69	25.4	5 Ene, 20 Ene	17, 15	< 70 km	8.4	53	54	44
	**L**	04 Dic	71	72	0	-	-	-	0.0	-	-	-
	**M**	04 Dic	69	70	22.3	24 Dic	20	< 10 km	2.3	0	71	14
**2008-09**	**N**	13 Nov	76	74	25.4*	23 Nov	10	< 70 km	*	69*	8*	18*
	**O**	07 Nov	71	70	16.9	26 Nov	18	< 10 km	1.1	35	31	22
	**P**	07 Nov	71	74	6.0	-	-	-	6.0	15	11	23
	**Q**	08 Nov	71	72	24.0*	-	-	-	*	16*	32*	33*
	**R**	09 Nov	71	72	6.9*	-	-	-	*	15*	24*	10*
	**Media (SD)**		**70.5 (2.0)**	**70.9 (2.3)**			**~17.5**		**2.7 (2.8) (n=11)**	**27.7 (18.6)**	**29.3 (25.4)**	**27.7 (22.3)**

* Instrumento dejó de transmitir antes de abandono de zona o cambio de modo comportamental
**Permaneció en modo ED la mayor parte del registro; distancias máximas calculadas usando los datos previos al último desove; tiempo total en ED y previo al último desove no calculados

Tabla 2. Uso del área marina protegida y zonas políticas por la tortuga golfina durante el

período entre-desove. Las abreviaciones empleadas son: Parque Nacional (PNM), Parque Transnacional propuesto (PTN).

	Zona (área total, km^2)	PNM (969.0)	PTN (2818.0)	Sólo zona de amortiguación (419.8)	Aguas gabonesas*	Aguas congolesas *
**80% (área total = 1267.7 km^2)**	km^2	565.3	1237.2	47.0	845.6	423.5
	% total ED registro	44.6	97.6	3.7	66.7	33.4
**100% DU (área total = 4414.8 km^2)**	km^2	841.4	2387.0	289.7	2048.2	2369.0
	% total ED registro	19.1	54.1	6.6	46.4	53.7

*El límite exacto entre Gabón y Congo no está bien definido, lo que resulta en una sobreposición entre las zonas calculadas

Tabla 3. Resumen del uso de las áreas marinas protegidas entre las posiciones promedio y re-muestreadas del MEE usando Distribución de Probabilidades (DU). Las abreviaciones empleadas son: Parque Nacional Mayumba (PNM), Parque Transnacional propuesto (PTN).

		Area Total	% DU total en PTN	% DU total en PNM	PNM	PTN	Zona amortiguación	ZEE gabonesa	ZEE congolesa
**80% DU: No. posiciones por celda (km^2)**	Promedio MEE	1267.7	97.6	44.6	565.3	1237.2	47.0	845.6	423.5
	Re-muestreo MEE	2368.8	88.0	33.0	781.9	2084.9	229.4	1523.3	846.0
	% diferencia	46.5	9.6	11.6	27.7	40.7	79.5	44.5	49.9
**100% DU: No. posiciones por celda (km^2)**	Promedio MEE	4414.8	54.1	19.1	841.4	2387.0	289.7	2048.2	2369.0
	Re-muestreo MME	20376.9	13.8	4.4	906.7	2818.0	419.8	7077.3	13299.6
	% diferencia	78.3	40.2	14.6	7.2	15.3	31.0	71.1	82.2

Figura 1. Todos los registros obtenidos con el Modelo de Estado Espacial (n=18) de tortugas golfina (*Lepidochelys olivacea*) instrumentadas en el Parque Nacional Mayumba. Los puntos rojos representan el modo comportamental entre-desove; los puntos grises representan el modo comportamental tránsito. La estrella indica el lugar de captura.

Figura 2. Distribución de Utilización (DU) de tortugas golfina (*Lepidochelys olivacea*) capturadas en el Parque Nacional Mayumba. La DU muestra que el Parque Transnacional propuesto abarca la mayor parte de la distribución de la especie. La estrella indica el lugar de captura.

Figura 3. Intervalos de confianza para el movimiento de la tortuga golfina (*Lepidochelys olivacea*) capturadas en el Parque Nacional Mayumba. La figura muestra el error exterior para la distribución de utilización del 80% para las posiciones promedio del MEE (verde claro) y posiciones re-muestreadas del MEE (verde oscuro). La estrella indica el lugar de captura.

Figura 4. Densidad de la Tortuga laúd (*Dermochelys coriacea*) (tomada de [33]) superpuesta con la distribución de utilización de la tortuga golfina (*Lepidochelys olivacea*). Se muestra las distribuciones similares y la efectividad de los límites del parque. La estrella indica el lugar de captura.
