# Supplementary material for: Using Satellite Tracking to Optimize Protection of Long-Lived Marine Species: Olive Ridley Sea Turtle Conservation in Central Africa
Source: PLoS One. 2011 May 11;6(5):e19905. doi: 10.1371/journal.pone.0019905 (PMC3092776; doi:10.1371/journal.pone.0019905)
Supplement: Text S2 — French translation of the abstract. (PDF) [file pone.0019905.s002.pdf]

L'emploi de la localisation par satellite pour optimiser la protection d'espèces marines à grande longévité : Préservation de la tortue olivâtre en Afrique centrale

Sara M. Maxwell[1*‡], Greg A. Breed[2], Barry A. Nickel[3], Junior Makanga-Bahouna[4], Edgard Pemo-Makaya[4], Richard J. Parnell[4], Angela Formia[5], Solange Ngouessono[6], Brendan J. Godley[7], Daniel P. Costa[2], Matthew J. Witt[7], Michael S. Coyne[7,8]

[1]Ocean Sciences Department, University of California Santa Cruz, Santa Cruz CA 95060 USA
[2]Department of Ecology and Evolutionary Biology, University of California Santa Cruz, Santa Cruz CA 95060 USA
[3]Center for Integrated Spatial Research, University of California Santa Cruz, Santa Cruz CA 95060 USA
[4]Wildlife Conservation Society, Parc National de Mayumba, Mayumba, Gabon
[5]Wildlife Conservation Society, Global Conservation Program, New York NY 10460 USA
[6]Agence Nationale des Parcs Nationaux, Libreville, Gabon
[7]Marine Turtle Research Group, Centre for Ecology and Conservation, University of Exeter – Cornwall Campus, Penyrn, Cornwall, TR10 9EZ UK
[8]SEATURTLE.org, Durham NC 27705, USA
*Corresponding author: smaxwell@ucsc.edu , +1 206 355 3249
‡Current address: Marine Conservation Institute, Long Marine Lab, 100 Shaffer Road, Santa Cruz CA 95060 USA

SOMMAIRE

Des mesures abordables pour la préservation des espèces à grande longévité demandent de se situer à l'intersection entre la protection des stades biologiquement importants de leur histoire naturelle et un cadre socio-économique réalisable. Pour la protection des adultes en âge de reproduction, nous nécessitons de connaissances sur les déplacements des animaux, la relation entre ces mouvements et les frontières politiques, et enfin d'analyses spatiales fiables des déplacements. Nous avons employé la localisation par satellite et un modèle spatio-temporel non stationnaire (‘switching state-space model’) pour déterminer les déplacements, entre leurs retours au nid, de tortues olivâtre (*Lepidochelys olivacea*) (n=18) en Afrique centrale au cours de deux saisons reproductives (2007-8 et 2008-9). Ces mouvements ont été analysés par rapport aux limites actuelles du parc, et aussi à un parc s'étendant entre le Gabon et la république du Congo dont la création future a été proposée, le but de tous deux étant la réduction de la capture involontaire de tortues de mer durant la pêche en mer. Nous avons aussi estimé l'intervalle de confiance autour du domaine vital calculé. Les tortues restaient souvent à l'intérieur d'un rayon de 30 km de leur lieu de nidation initial avant de le quitter pour des zones d'alimentation éloignées. Seulement 44,6% des zones de haute densité se trouvaient à l'intérieur du parc actuel, alors que le parc proposé à cheval des frontières en couvrirait les 97,6%. Bien que l'origine des animaux suivis se trouvât au Gabon, les tortues étaient dans les eaux congolaises pendant plus de la moitié des intervalles entre les retours au nid (53,7%). Cette constatation souligne le

besoin de coopération internationale et offre un soutien scientifique à la proposition de créer un parc à travers les frontières. Cette première étude détaillée des déplacements des tortues olivâtres qui s'isolent pendant leur nidation suggère des possibilités de mesures abordables de préservation des individus femelles au nid, et aussi en d'autres lieux de nidation solitaire dans le reste du monde. Nos résultats nous permettent de définir un cadre pour la protection au meilleur coût des espèces à grande longévité en utilisant la télémétrie par satellite comme instrument principal.

NOTE : S'il vous plaît écrire à Sara Maxwell (smaxwell@ucsc.edu) pour l'article entier en français.
